# The minimum number of rotations about two axes for constructing an arbitrarily fixed rotation

**DOI:** 10.1098/rsos.140145

**Published:** 2014-11-26

**Authors:** Mitsuru Hamada

**Affiliations:** Quantum Information Science Research Center, Tamagawa University, Tamagawa-gakuen 6-chome, Machida, Tokyo 194-8610, Japan

**Keywords:** SU(2), SO(3), rotation

## Abstract

For any pair of three-dimensional real unit vectors $\hat{m}$ and $\hat{n}$ with $|{\hat{m}}^{T}\hat{n}|<1$ and any rotation *U*, let $N_{\hat{m},\hat{n}}(U)$ denote the least value of a positive integer *k* such that *U* can be decomposed into a product of *k* rotations about either $\hat{m}$ or $\hat{n}$. This work gives the number $N_{\hat{m},\hat{n}}(U)$ as a function of *U*. Here, a rotation means an element *D* of the special orthogonal group *SO*(3) or an element of the special unitary group *SU*(2) that corresponds to *D*. Decompositions of *U* attaining the minimum number $N_{\hat{m},\hat{n}}(U)$ are also given explicitly.

## 2. Introduction

In this work, an issue on optimal constructions of rotations in the Euclidean space $R^{3}$, under some restriction, is addressed and solved. By a rotation or rotation matrix, we usually mean an element of the special orthogonal group *SO*(3). However, we follow the custom, in quantum physics, to call not only an element of *SO*(3) but also that of the special unitary group *SU*(2) a rotation. This is justified by the well-known homomorphism from *SU*(2) onto *SO*(3) (§2.4). Given a pair of three-dimensional real unit vectors $\hat{m}$ and $\hat{n}$ with $|{\hat{m}}^{T}\hat{n}|<1$, where ${\hat{m}}^{T}$ denotes the transpose of $\hat{m}$, let $N_{\hat{m},\hat{n}}(A)$ denote the least value of a positive integer *k* such that any rotation in A can be decomposed into (constructed as) a product of *k* rotations about either $\hat{m}$ or $\hat{n}$, where A=SU(2),SO(3). It is known that $N_{\hat{m},\hat{n}}(\mathrm{SO}(3))=N_{\hat{m},\hat{n}}(\mathrm{SU}(2))=\lceil \pi /\arccos|{\hat{m}}^{T}\hat{n}|\rceil +1$ for any pair of three-dimensional real unit vectors $\hat{m}$ and $\hat{n}$ with $|{\hat{m}}^{T}\hat{n}|<1$ [1,2].

Then, a natural question arises: What is the least value, $N_{\hat{m},\hat{n}}(U)$, of a positive integer *k* such that an arbitrarily fixed rotation *U* can be decomposed into a product of *k* rotations about either $\hat{m}$ or $\hat{n}$? In this work, the minimum number $N_{\hat{m},\hat{n}}(U)$ is given as an explicit function of *U*, where *U* is expressed in terms of parameters known as Euler angles [3,4]. Moreover, optimal, that is minimum-achieving, decompositions (constructions) of any fixed element *U*∈*SU*(2) are presented explicitly.

In this work, not only explicit constructions but also simple inequalities on geometric quantities, which directly show lower bounds on the number of constituent rotations, will be presented. Remarkably, the proposed explicit constructions meet the obtained lower bounds, which shows both the optimality of the constructions and the tightness of the bounds.

The results in this work were obtained before the author came to know Lowenthal's formula on $N_{\hat{m},\hat{n}}(\mathrm{SO}(3))$ [1,2] and a related result [5]. Prior to this work, the work by D'Alessandro [5] has treated the issue of determining $N_{\hat{m},\hat{n}}(D)$, *D*∈*SO*(3). That interesting result [5], however, gave $N_{\hat{m},\hat{n}}(D)$, *D*∈*SO*(3), only algorithmically (with the largest index of a sequence of real numbers with some property). The distinctive features of this work include the following: $N_{\hat{m},\hat{n}}(U)$ is given in terms of an explicit function of parameters of *U*∈*SU*(2); explicit optimal decompositions are presented; and this work's results on $N_{\hat{m},\hat{n}}(U)$ imply Lowenthal's formula on $N_{\hat{m},\hat{n}}(\mathrm{SO}(3))$ in a consistent self-contained manner.^1^

Regarding another direction of related research, we remark that $N_{\hat{m},\hat{n}}(A)$ is known as the order of (uniform) generation of the Lie group A, and this notion has been extended to other Lie groups. The interested reader is referred to relatively extensive treatments on uniform generation [6,7], where one would find that even determining the order $N_{\hat{m},\hat{n}}(\mathrm{SO}(3))$ needs a special proof (see [1,2] and [7, Appendix]).

Detailed elementary arguments below would help us dispel some confusions related to $N_{\hat{m},\hat{n}}(\mathrm{SU}(2))$ often found in textbooks on quantum computation. There, not to mention the ignorance of the fact $N_{\hat{m},\hat{n}}(\mathrm{SU}(2))=\lceil \pi /\arccos|{\hat{m}}^{T}\hat{n}|\rceil +1$, a wrong statement equivalent to saying that $N_{\hat{m},\hat{n}}(\mathrm{SU}(2))$ were, at most, *three*, regardless of the choice of non-parallel vectors $\hat{m}$ and $\hat{n}$, is observed.

Regarding physics, this work has been affected by the issue of constructing an arbitrary unitary operator on a Hilbert space discussed in quantum physics [8]. This is relevant to universal gates for quantum computation [9]. In this context, requiring the availability of rotations about a pair of exactly orthogonal axes seems too idealistic. For example, consider a Hamiltonian *H* of a quantum system represented by $C^{2}$, and note that *H* determines the axis of the rotations ${\left[c(t)\right]}^{-1}\exp(-itH)\in \mathrm{SU}(2)$, t∈R, where *c*(*t*) is a square root of detexp⁡(−itH). (Often, although not always, differences of unitary matrices (evolutions) up to scalar multiples are ignorable.) Thus, explicit decompositions attaining the minimum $N_{\hat{m},\hat{n}}(U)$ of an arbitrary rotation *U* for the generic vectors $\hat{m}$ and $\hat{n}$ will be useful. For applications to control, the reader is referred to D'Alessandro [5] and references therein.

This paper is organized as follows. After giving preliminaries in §2, the main theorem establishing $N_{\hat{m},\hat{n}}(U)$ and explicit constructions of rotations are presented in §3. Then, inequalities that show limits on constructions are presented in §4. The proofs of the results of this work are presented in §5. Section 6 contains the conclusion. Several arguments are relegated to appendices.

## 3. Preliminaries and a known result

### 3.1 Definitions

The notation to be used includes the following: N denotes the set of strictly positive integers; $S^{2}=\{\hat{v}\in R^{3}|∥\hat{v}∥=1\}$, where $∥\hat{v}∥=\sqrt{v_{x}^{2}+v_{y}^{2}+v_{z}^{2}}$ for $\hat{v}={\left(v_{x},v_{y},v_{z}\right)}^{T}$; ⌈*x*⌉ denotes the smallest integer not less than x∈R. As usual, arccos⁡x∈[0,π] and arcsin⁡x∈[−π/2,π/2] for *x*∈[−1,1]. The Hermitian conjugate of a matrix *U* is denoted by *U*^†^.

Throughout, *I* denotes the 2×2 identity matrix; *X*, *Y* and *Z* denote the following Pauli matrices:

$$X=\left(\begin{array}{cc} 0 & 1\\ 1 & 0 \end{array}\right),\;Y=\left(\begin{array}{cc} 0 & -i\\ i & 0 \end{array}\right)\;\mathrm{and}\;Z=\left(\begin{array}{cc} 1 & 0\\ 0 & -1 \end{array}\right).$$

We shall work with a matrix

3.1 $$R_{\hat{v}}(\theta ):=\left(\cos\frac{\theta }{2}\right)I-i\left(\sin\frac{\theta }{2}\right)(v_{x}X+v_{y}Y+v_{z}Z),$$

where $\hat{v}={\left(v_{x},v_{y},v_{z}\right)}^{T}\in S^{2}$ and θ∈R. This represents the rotation about $\hat{v}$ by angle *θ* (through the homomorphism in §2.4). In particular, for $\hat{y}={\left(0,1,0\right)}^{T}$ and $\hat{z}={\left(0,0,1\right)}^{T}$, we put

$$R_{y}(\theta ):=R_{\hat{y}}(\theta )=\left(\begin{array}{cc} \cos\frac{\theta }{2} & -\sin\frac{\theta }{2}\\ \sin\frac{\theta }{2} & \cos\frac{\theta }{2} \end{array}\right)\;\text{and}\;R_{z}(\theta ):=R_{\hat{z}}(\theta )=\left(\begin{array}{cc} e^{-i(\theta /2)} & 0\\ 0 & e^{i(\theta /2)} \end{array}\right).$$

For $\hat{m},\hat{n}\in S^{2}$ with $|{\hat{m}}^{T}\hat{n}|<1$, we define the following:

3.2 $$N_{\hat{m},\hat{n}}(U):=\min\{j\in N|\exists V_{1},\ldots ,V_{j}\in R_{\hat{m}}\cup R_{\hat{n}},\;U=V_{1}\ldots V_{j}\}$$

for *U*∈*SU*(2), where $R_{\hat{v}}:=\{R_{\hat{v}}(\theta )|\theta \in R\}$, and

3.3 $$N_{\hat{m},\hat{n}}:=N_{\hat{m},\hat{n}}(\mathrm{SU}(2)):=\min\{k\in N|\forall U\in \mathrm{SU}(2),\;N_{\hat{m},\hat{n}}(U)\leq k\}.$$

Using the homomorphism *F* from SU(2) onto SO(3) to be defined in §3.4, we put ${\hat{R}}_{\hat{v}}:=\{F(R_{\hat{v}}(\theta ))|\theta \in R\}$. We extend the definition of $N_{\hat{m},\hat{n}}$ to SO(3):

3.4 $$N_{\hat{m},\hat{n}}(D):=\min\{j\in N|\exists A_{1},\ldots ,A_{j}\in {\hat{R}}_{\hat{m}}\cup {\hat{R}}_{\hat{n}},\;D=A_{1}\cdots A_{j}\}$$

for *D*∈SO(3) and

3.5 $$N_{\hat{m},\hat{n}}(\mathrm{SO}(3)):=\min\{k\in N|\forall D\in \mathrm{SO}(3),\;N_{\hat{m},\hat{n}}(D)\leq k\}.$$

### 3.2 The maximum of the minimum number of constituent rotations over all target rotations

This work's results lead to an elementary self-contained proof of the following known theorem (appendix F).

**Theorem 3.1 (Lowenthal [1,2])**

*For any* $\hat{m},\hat{n}\in S^{2}$ *with* $|{\hat{m}}^{T}\hat{n}|<1,$

$$N_{\hat{m},\hat{n}}(\mathrm{SO}(3))=N_{\hat{m},\hat{n}}(\mathrm{SU}(2))=\left\lceil \frac{\pi }{\arccos|{\hat{m}}^{T}\hat{n}|}\right\rceil +1.$$

### 3.3 Parametrizations of the elements in SU(2)

The following lemma presents a well-known parametrization of SU(2) elements.

**Lemma 3.2**

*For any element* *U*∈SU(2), *there exist some* α,γ∈R *and* *β*∈[0,*π*] *such that*

3.6 $$U=\left(\begin{array}{cc} e^{-i((\gamma +\alpha )/2)}\cos\frac{\beta }{2} & -e^{i((\gamma -\alpha )/2)}\sin\frac{\beta }{2}\\ e^{-i((\gamma -\alpha )/2)}\sin\frac{\beta }{2} & e^{i((\gamma +\alpha )/2)}\cos\frac{\beta }{2} \end{array}\right)=R_{z}(\alpha )R_{y}(\beta )R_{z}(\gamma ).$$

The parameters *α*,*β* and *γ* in this lemma are often called Euler angles.^2^ The lemma can be rephrased as follows: any matrix in SU(2) can be written as

3.7 $$\left(\begin{array}{cc} a & b\\ -b^{*} & a^{*} \end{array}\right)$$

with some complex numbers *a* and *b* such that |*a*|^2^+|*b*|^2^=1 [3]. Hence, any matrix in SU(2) can be written as

3.8 $$\left(\begin{array}{cc} w+iz & y+ix\\ -y+ix & w-iz \end{array}\right)=wI+i(xX+yY+zZ)$$

with some real numbers *x*,*y*,*z* and *w* such that *w*^2^+*x*^2^+*y*^2^+*z*^2^=1. Take a real number *θ* such that cos⁡(θ/2)=w and $\sin(\theta /2)=\sqrt{1-w^{2}}=\sqrt{x^{2}+y^{2}+z^{2}}$; write *x*, *y* and *z* as $x=-v_{x}\sin(\theta /2),y=-v_{y}\sin(\theta /2)$ and $z=-v_{z}\sin(\theta /2)$, where $v_{x},v_{y},v_{z}\in R$ and $v_{x}^{2}+v_{y}^{2}+v_{z}^{2}=1$. Thus, using real numbers $\theta ,v_{x},v_{y},v_{z}\in R$ with $v_{x}^{2}+v_{y}^{2}+v_{z}^{2}=1$, any matrix in SU(2) can be written as

$$\left(\cos\frac{\theta }{2}\right)I-i\left(\sin\frac{\theta }{2}\right)(v_{x}X+v_{y}Y+v_{z}Z),$$

which is nothing but $R_{\hat{v}}(\theta )$ in (3.1).

### 3.4 Homomorphism from SU(2) onto SO(3)

For *U*∈SU(2), we denote by *F*(*U*) the matrix of the linear transformation on $R^{3}$ that sends (*x*,*y*,*z*)^*T*^ to (*x*′,*y*′,*z*′)^*T*^ through^3^

3.9 $$U(xX+yY+zZ)U^{†}=x^{\prime }X+y^{\prime }Y+z^{\prime }Z.$$

Namely, for any ${\left(x,y,z\right)}^{T},{\left(x^{\prime },y^{\prime },z^{\prime }\right)}^{T}\in R^{3}$ with (3.9),

$$\left(\begin{array}{c} x^{\prime }\\ y^{\prime }\\ z^{\prime } \end{array}\right)=F(U)\left(\begin{array}{c} x\\ y\\ z \end{array}\right).$$

We also define

3.10 $${\hat{R}}_{\hat{v}}(\theta ):=F(R_{\hat{v}}(\theta ))\;\mathrm{for}\;\hat{v}\in S^{2},\theta \in R.$$

### 3.5 Generic orthogonal axes and coordinate axes

Lemma 3.2 can be generalized as follows.

**Lemma 3.3**

*Let* $\hat{l},\hat{m}\in S^{2}$ *be vectors with* $\hat{l}{\;}^{T}\hat{m}=0$. *Then, for any* *V* ∈SU(2), *there exist some* α,γ∈R *and* *β*∈[0,*π*] *such that*

3.11 $$V=R_{\hat{m}}(\alpha )R_{\hat{l}}(\beta )R_{\hat{m}}(\gamma ).$$

**Proof**

As *F* is onto SO(3), there exists an element *U*∈SU(2) such that $\hat{l}=F(U){\left(0,1,0\right)}^{T}$ and $\hat{m}=F(U){\left(0,0,1\right)}^{T}$.^4^ With this element *U*, some α,γ∈R and some *β*∈[0,*π*], write *U*^†^*V* *U*=*R*_*z*_(*α*)*R*_*y*_(*β*)*R*_*z*_(*γ*) in terms of the parametrization (3.6). Then, since $UR_{z}(\alpha )U^{†}=R_{\hat{m}}(\alpha )$, $UR_{y}(\beta )U^{†}=R_{\hat{l}}(\beta )$ and $UR_{z}(\gamma )U^{†}=R_{\hat{m}}(\gamma )$, we obtain (3.11). ▪

We also have the following lemma, which is easy but worth recognizing.

**Lemma 3.4**

*Let arbitrary* κ,ν∈N, ${\hat{u}}_{1},\ldots ,{\hat{u}}_{\kappa },{\hat{v}}_{1},\ldots ,{\hat{v}}_{\nu }\in S^{2}$ *and* *U*∈SU(2) *be given. Put* ${\hat{u}}_{1}^{\prime }=F(U){\hat{u}}_{1},\ldots ,{\hat{u}}_{\kappa }^{\prime }=F(U){\hat{u}}_{\kappa },{\hat{v}}_{1}^{\prime }=F(U){\hat{v}}_{1},\ldots$ *and* ${\hat{v}}_{\nu }^{\prime }=F(U){\hat{v}}_{\nu }$. *Then, for any* $\theta_{1},\ldots ,\theta_{\kappa },\phi_{1},\ldots \phi_{\nu }\in R,$

$$R_{{\hat{u}}_{1}}(\theta_{1})\cdots R_{{\hat{u}}_{\kappa }}(\theta_{\kappa })=R_{{\hat{v}}_{1}}(\phi_{1})\cdots R_{{\hat{v}}_{\nu }}(\phi_{\nu })$$

*if and only if* (*iff*)

$$R_{{\hat{u}}_{1}^{\prime }}(\theta_{1})\cdots R_{{\hat{u}}_{\kappa }^{\prime }}(\theta_{\kappa })=R_{{\hat{v}}_{1}^{\prime }}(\phi_{1})\cdots R_{{\hat{v}}_{\nu }^{\prime }}(\phi_{\nu }).$$

**Proof**

This readily follows from $UR_{{\hat{u}}_{j}}(\theta_{j})U^{†}=R_{{\hat{u}}_{j}^{\prime }}(\theta_{j})$ and $UR_{{\hat{v}}_{j}}(\phi_{j})U^{†}=R_{{\hat{v}}_{j}^{\prime }}(\phi_{j})$. ▪

## 4. The minimum numbers of constituent rotations and optimal constructions of an arbitrary rotation

Here, we present the result establishing $N_{\hat{m},\hat{n}}(U)$ with needed definitions.

**Definition 4.1**

For $\hat{v}\in S^{2}$ and

4.1 $$U=\left(\begin{array}{cc} w+iz & y+ix\\ -y+ix & w-iz \end{array}\right)=wI+i(xX+yY+zZ)\in \mathrm{SU}(2),$$

where w,x,y,z∈R are parameters to express *U* uniquely, $b(\hat{v},U)$ is defined by

4.2 $$b(\hat{v},U):=|(x,y,z)\hat{v}|.$$

**Definition 4.2**

Functions $f:R^{3}\to [0,\pi ]$ and $g:R^{2}\times (0,\pi /2]\to N$ are defined by

$$f(\alpha ,\beta ,\delta ):=2\arccos\sqrt{\cos^{2}\frac{\beta }{2}\cos^{2}\frac{\delta }{2}+\sin^{2}\frac{\beta }{2}\sin^{2}\frac{\delta }{2}+2\cos\alpha \sin\frac{\beta }{2}\sin\frac{\delta }{2}\cos\frac{\beta }{2}\cos\frac{\delta }{2}}$$

and

$$g(\alpha ,\beta ,\delta ):=\left\{ \begin{array}{ll} 2\left\lceil \frac{f(\alpha ,\beta ,\delta )}{2\delta }+\frac{1}{2}\right\rceil & \text{if }f(\alpha ,\beta ,\delta )\geq \delta \\ 4 & \text{otherwise.} \end{array}\right.$$

**Theorem 4.3**

*For any* $\hat{m},\hat{n}\in S^{2}$ *with* ${\hat{m}}^{T}\hat{n}\in [0,1),$ α,γ∈R *and* β∈[0,π], *if*

$$b(\hat{m},U_{\alpha ,\beta ,\gamma }^{\hat{m},\hat{l}})\geq b(\hat{n},U_{\alpha ,\beta ,\gamma }^{\hat{m},\hat{l}}),$$

*then*

$$N_{\hat{m},\hat{n}}(F(U_{\alpha ,\beta ,\gamma }^{\hat{m},\hat{l}}))=N_{\hat{m},\hat{n}}(U_{\alpha ,\beta ,\gamma }^{\hat{m},\hat{l}})=\min\left\{2\left\lceil \frac{\beta }{2\delta }\right\rceil +1,g(\alpha ,\beta ,\delta ),g(\gamma ,-\beta ,\delta )\right\},$$

*where* $\delta =\arccos{\hat{m}}^{T}\hat{n}\in (0,\pi /2],$ $\hat{l}=∥\hat{m}\times \hat{n}{∥}^{-1}\hat{m}\times \hat{n}$ *and*

$$U_{\alpha ,\beta ,\gamma }^{\hat{m},\hat{l}}:=R_{\hat{m}}(\alpha )R_{\hat{l}}(\beta )R_{\hat{m}}(\gamma ).$$

Note that there is no loss of generality in assuming $b(\hat{m},U_{\alpha ,\beta ,\gamma }^{\hat{m},\hat{l}})\geq$ $b(\hat{n},U_{\alpha ,\beta ,\gamma }^{\hat{m},\hat{l}})$, but also note that *α*,*β* and *γ* vary, in general, if $\hat{m}$ and $\hat{n}$ are interchanged.

We give two constructions or decompositions, which will turn out to attain the minimum number $N_{\hat{m},\hat{n}}(U_{\alpha ,\beta ,\gamma }^{\hat{m},\hat{l}})$ in the theorem.

**Proposition 4.4**

*Given arbitrary* $\hat{m},\hat{n}\in S^{2}$ *with* ${\hat{m}}^{T}\hat{n}\in [0,1),$ α,γ∈R *and* *β*∈[0,*π*], *put*

4.3 $$\delta =\arccos{\hat{m}}^{T}\hat{n}\in \left(0,\frac{\pi }{2}\right]$$

*and*

$$\hat{l}=∥\hat{m}\times \hat{n}{∥}^{-1}\hat{m}\times \hat{n}.$$

*Then, for any* k∈N *and* *β*_1_,…,*β*_*k*_∈(0,2*δ*] *satisfying*

4.4 $$\beta =\beta_{1}+\cdots +\beta_{k},$$

*there exist some* $\alpha_{j},\gamma_{j},\theta_{j}\in R$ *such that*

4.5 $$R_{\hat{l}}(\beta_{j})=R_{\hat{m}}(-\alpha_{j})R_{\hat{n}}(\theta_{j})R_{\hat{m}}(-\gamma_{j})$$

*for* *j*=1,…,*k*. *For these parameters, it holds that*

4.6 $$\begin{array}{rl} & R_{\hat{m}}(\alpha )R_{\hat{l}}(\beta )R_{\hat{m}}(\gamma )\\ & \;=R_{\hat{m}}(\alpha -\alpha_{1})R_{\hat{n}}(\theta_{1})R_{\hat{m}}(-\gamma_{1}-\alpha_{2})R_{\hat{n}}(\theta_{2})R_{\hat{m}}(-\gamma_{2}-\alpha_{3})R_{\hat{n}}(\theta_{3})\cdots \\ & \;\cdot R_{\hat{m}}(-\gamma_{k-1}-\alpha_{k})R_{\hat{n}}(\theta_{k})R_{\hat{m}}(-\gamma_{k}+\gamma ). \end{array}$$

**Remark 4.5**

The least value of *k* such that (4.4) holds for some *β*_1_,…,*β*_*k*_∈ (0,2*δ*] is ⌈*β*/(2*δ*)⌉.^5^ Hence, this proposition gives a decomposition of an arbitrary element $U=R_{\hat{m}}(\alpha )R_{\hat{l}}(\beta )R_{\hat{m}}(\gamma )\in \mathrm{SU}(2)$ into the product of 2⌈*β*/(2*δ*)⌉+1 rotations.^6^

**Remark 4.6**

For β,δ∈R with 0≤*β*/2≤*δ*≤*π*/2, *δ*≠0, and t∈R, let

$$H_{t}(\beta ,\delta ):=\left\{ \begin{array}{ll} 0 & \text{if }\frac{\beta }{2}<\delta =\frac{\pi }{2}\\ t & \text{if }\frac{\beta }{2}=\delta =\frac{\pi }{2}\\ \arcsin\frac{\tan(\beta /2)}{\tan\delta } & \text{otherwise.} \end{array}\right.$$

Then, an explicit instance of the set of parameters *α*_*j*_, *γ*_*j*_ and *θ*_*j*_ for which (4.5) holds is given by (*α*_*j*_,*γ*_*j*_,*θ*_*j*_)^*T*^=*σ*_*t*_*j*__(*β*_*j*_,*δ*), where

4.7 $$\sigma_{t}(\beta ,\delta ):=\left(\begin{array}{c} H_{t}(\beta ,\delta )-\frac{\pi }{2}\\ H_{t}(\beta ,\delta )+\frac{\pi }{2}\\ 2\arcsin\frac{\sin(\beta /2)}{\sin\delta } \end{array}\right)$$

and $t_{j}\in R$ can be chosen arbitrarily, *j*=1,…,*k*. (These make (4.6) hold.)

**Proposition 4.7**

*Given any* $\hat{m},\hat{n}\in S^{2}$ *with* ${\hat{m}}^{T}\hat{n}\in [0,1),$ *put* $\delta =\arccos{\hat{m}}^{T}\hat{n}\in (0,\pi /2]$ *and* $\hat{l}=∥\hat{m}\times \hat{n}{∥}^{-1}\hat{m}\times \hat{n}$. *For an arbitrary* *U*∈SU(2), *choose parameters* $\alpha^{\prime },\gamma^{\prime }\in R$ *and* *β*′∈[0,*π*] *such that*

4.8 $$R_{\hat{l}}(-\delta )U=R_{\hat{m}}(\alpha^{\prime })R_{\hat{l}}(\beta^{\prime })R_{\hat{m}}(\gamma^{\prime }).$$

*Then*,

4.9 $$U=R_{\hat{n}}(\alpha^{\prime })R_{\hat{l}}(\beta^{\prime }+\delta )R_{\hat{m}}(\gamma^{\prime }).$$

*Furthermore, for any* $k^{\prime }\in N$ *and* *β*′_1_,…,*β*′_*k*′_∈(0,2*δ*] *satisfying*

4.10 $$\beta^{\prime }+\delta =\beta_{1}^{\prime }+\cdots +\beta_{k^{\prime }}^{\prime },$$

*there exist some* $\alpha_{j}^{\prime },\gamma_{j}^{\prime },\theta_{j}^{\prime }\in R$ *such that*

4.11 $$R_{\hat{l}}(\beta_{j}^{\prime })=R_{\hat{m}}(-\alpha_{j}^{\prime })R_{\hat{n}}(\theta_{j}^{\prime })R_{\hat{m}}(-\gamma_{j}^{\prime })$$

*for* *j*=1,…,*k*′. *For these parameters, it holds that*

4.12 $$\begin{array}{rl} U & =R_{\hat{n}}(\alpha^{\prime })R_{\hat{m}}(-\alpha_{1}^{\prime })R_{\hat{n}}(\theta_{1}^{\prime })R_{\hat{m}}(-\gamma_{1}^{\prime }-\alpha_{2}^{\prime })R_{\hat{n}}(\theta_{2}^{\prime })R_{\hat{m}}(-\gamma_{2}^{\prime }-\alpha_{3}^{\prime })R_{\hat{n}}(\theta_{3}^{\prime })\cdots \\ & \;\cdot R_{\hat{m}}(-\gamma_{k^{\prime }-1}^{\prime }-\alpha_{k^{\prime }}^{\prime })R_{\hat{n}}(\theta_{k^{\prime }}^{\prime })R_{\hat{m}}(-\gamma_{k^{\prime }}^{\prime }+\gamma^{\prime }). \end{array}$$

**Remark 4.8**

The least value of *k*′ such that (4.10) holds for some *β*′_1_,…,*β*′_*k*′_∈ (0,2*δ*] is ⌈(*β*′+*δ*)/(2*δ*)⌉=⌈*β*′/(2*δ*)+1/2⌉. Moreover, if *β*′≥*δ* and *k*′=⌈*β*′/(2*δ*)+1/2⌉, the parameter *α*′_1_ can be chosen so that it satisfies *α*′_1_=0 as well as (4.11) and (4.12). Hence, when *β*′≥*δ*, this proposition and the fact just mentioned give a decomposition of an arbitrary element $U=R_{\hat{n}}(\alpha^{\prime })R_{\hat{l}}(\beta^{\prime }+\delta )R_{\hat{m}}(\gamma^{\prime })\in \mathrm{SU}(2)$ into the product of $2\lceil \beta^{\prime }/(2\delta )+\frac{1}{2}\rceil$ rotations, and when *β*′<*δ*, a decomposition of *U* into the product of four rotations.

**Remark 4.9**

An explicit instance of the set of parameters *α*′_*j*_,*γ*′_*j*_ and *θ*′_*j*_, *j*=1,…,*k*′, for which (4.11) and (4.12) hold is given by (*α*′_*j*_,*γ*′_*j*_,*θ*′_*j*_)^*T*^= *σ*_*t*_*j*__(*β*′_*j*_,*δ*), where $t_{j}\in R$ can be chosen arbitrarily, *j*=1,…,*k*′.

## 5. Limits on constructions

In order to bound $N_{\hat{m},\hat{n}}(D)$, etc., from below, we use the geodesic metric on the unit sphere *S*^2^, which is denoted by *d*. Specifically,

5.1 $$d(\hat{u},\hat{v}):=\arccos{\hat{u}}^{T}\hat{v}\in [0,\pi ]$$

for $\hat{u},\hat{v}\in S^{2}$. This is the length of the geodesic connecting $\hat{u}$ and $\hat{v}$ on *S*^2^. We have the following lemma. (Recall we have put ${\hat{R}}_{\hat{v}}(\theta )=F(R_{\hat{v}}(\theta ))$.)

**Lemma 5.1**

*Let* $\hat{n},\hat{m}$ *be arbitrary vectors in* *S*^2^ *with* $\delta =d(\hat{m},\hat{n})=\arccos{\hat{m}}^{T}\hat{n}\in (0,\pi ]$. *Then, for any* k∈N *and* $\phi_{1},\ldots ,\phi_{2k}\in R,$ *the following inequalities hold*:

5.2 $$\begin{array}{rl} & d({\hat{R}}_{\hat{m}}(\phi_{2k-1}){\hat{R}}_{\hat{n}}(\phi_{2k-2})\cdots {\hat{R}}_{\hat{m}}(\phi_{3}){\hat{R}}_{\hat{n}}(\phi_{2}){\hat{R}}_{\hat{m}}(\phi_{1})\hat{m},\hat{m})\leq 2(k-1)\delta , \end{array}$$

5.3 $$\begin{array}{rl} & d({\hat{R}}_{\hat{m}}(\phi_{2k-1}){\hat{R}}_{\hat{n}}(\phi_{2k-2})\cdots {\hat{R}}_{\hat{m}}(\phi_{3}){\hat{R}}_{\hat{n}}(\phi_{2}){\hat{R}}_{\hat{m}}(\phi_{1})\hat{m},\hat{n})\leq (2k-1)\delta , \end{array}$$

5.4 $$\begin{array}{rl} & d({\hat{R}}_{\hat{n}}(\phi_{2k}){\hat{R}}_{\hat{m}}(\phi_{2k-1})\cdots {\hat{R}}_{\hat{m}}(\phi_{3}){\hat{R}}_{\hat{n}}(\phi_{2}){\hat{R}}_{\hat{m}}(\phi_{1})\hat{m},\hat{n})\leq (2k-1)\delta \end{array}$$

5.5 $$\begin{array}{rl} & \text{and}\;d({\hat{R}}_{\hat{n}}(\phi_{2k}){\hat{R}}_{\hat{m}}(\phi_{2k-1})\cdots {\hat{R}}_{\hat{m}}(\phi_{3}){\hat{R}}_{\hat{n}}(\phi_{2}){\hat{R}}_{\hat{m}}(\phi_{1})\hat{m},\hat{m})\leq 2k\delta . \end{array}$$

This can be shown easily by induction on *k* using the triangle inequality for *d*. In what follows, (5.2) and (5.4) will be used in the following forms:

5.6 $$2\left\lceil \frac{d(D\hat{m},\hat{m})}{2\delta }\right\rceil +1\leq 2k-1\;\text{and}\;2\left\lceil \frac{d(D^{\prime }\hat{m},\hat{n})}{2\delta }+\frac{1}{2}\right\rceil \leq 2k.$$

These bounds hold when *D* and *D*′∈SO(3) equal the product of 2*k*−1 rotations and that of 2*k* rotations, respectively, in lemma 5.1 (since *k* is an integer). It will turn out that these bounds are tight.

## 6. Proof of the results

### 6.1 Structure of the proof

Here, the structure of the whole proof of the results in this work is described. Theorem 4.3 is obtained as a consequence of lemma 6.2 to be presented. The constructive half of lemma 6.2 is due to propositions 4.4 and 4.7. The other half of lemma 6.2, related to limits on constructions, is due to lemma 5.1. Theorem 3.1 is derived from theorem 4.3 in appendix F.

### 6.2 Proof of propositions 4.4 and 4.7

The following lemma is fundamental to the results in this work.

**Lemma 6.1**

*For any* β,θ∈R *and for any* $\hat{u},\hat{l},\hat{m}\in S^{2}$ *such that* $\hat{l}{\;}^{T}\hat{m}=0,$ *the following two conditions are equivalent*.

I. *There exist some* α,γ∈R *such that*
  6.1 $$R_{\hat{u}}(\theta )=R_{\hat{m}}(\alpha )R_{\hat{l}}(\beta )R_{\hat{m}}(\gamma ).$$
II. $\sqrt{1-{\left({\hat{m}}^{T}\hat{u}\right)}^{2}}|\sin(\theta /2)|=|\sin(\beta /2)|$.

**Proof**

(1) Take an element *U*∈SU(2) such that

6.2 $$\hat{l}=F(U){\left(0,1,0\right)}^{T}\;\text{and}\;\hat{m}=F(U){\left(0,0,1\right)}^{T},$$

and put $\hat{v}={\left(v_{x},v_{y},v_{z}\right)}^{T}$ for the parameters *v*_*x*_,*v*_*y*_ and *v*_*z*_ such that

6.3 $$\hat{u}=v_{x}\hat{l}\times \hat{m}+v_{y}\hat{l}+v_{z}\hat{m}.$$

Then, owing to lemma 3.4, (6.1) holds iff

6.4 $$R_{\hat{v}}(\theta )=R_{z}(\alpha )R_{y}(\beta )R_{z}(\gamma ).$$

(2) A direct calculation shows

6.5 $$\begin{array}{rl} R_{z}(\alpha )R_{y}(\beta )R_{z}(\gamma ) & =\cos\frac{\beta }{2}\cos\frac{\gamma +\alpha }{2}I-i\sin\frac{\beta }{2}\sin\frac{\gamma -\alpha }{2}X\\ & \;-i\sin\frac{\beta }{2}\cos\frac{\gamma -\alpha }{2}Y-i\cos\frac{\beta }{2}\sin\frac{\gamma +\alpha }{2}Z. \end{array}$$

Hence, (6.4) is equivalent to

6.6 $$\begin{array}{rl} \cos\frac{\theta }{2} & =\cos\frac{\beta }{2}\cos\frac{\gamma +\alpha }{2}, \end{array}$$

6.7 $$\begin{array}{rl} v_{x}\sin\frac{\theta }{2} & =\sin\frac{\beta }{2}\sin\frac{\gamma -\alpha }{2}, \end{array}$$

6.8 $$\begin{array}{rl} v_{y}\sin\frac{\theta }{2} & =\sin\frac{\beta }{2}\cos\frac{\gamma -\alpha }{2} \end{array}$$

6.9 $$\begin{array}{rl} \text{and}\;v_{z}\sin\frac{\theta }{2} & =\cos\frac{\beta }{2}\sin\frac{\gamma +\alpha }{2}. \end{array}$$

(3) We shall prove I ⇒ II. On each side of (6.7) and (6.8), squaring and summing the resultant pair, we have

6.10 $$\sqrt{1-v_{z}^{2}}\left|\sin\frac{\theta }{2}\right|=\left|\sin\frac{\beta }{2}\right|.$$

(Equations (6.6) and (6.9) also imply (6.10) similarly.) But (6.10) implies II in view of (6.3).

(4) Next, we shall prove II ⇒ I.

Transforming (*α*,*β*) into (*η*,*ζ*), where the two pairs are related by

6.11 $$\eta =\frac{\gamma +\alpha }{2}\;\text{and}\;\zeta =\frac{\gamma -\alpha }{2},$$

we see, from paragraphs (1) and (2), that I is equivalent to the following condition: There exist some η,ζ∈R such that

6.12 $$\begin{array}{rl} \cos\frac{\theta }{2} & =\cos\frac{\beta }{2}\cos\eta , \end{array}$$

6.13 $$\begin{array}{rl} v_{x}\sin\frac{\theta }{2} & =\sin\frac{\beta }{2}\sin\zeta , \end{array}$$

6.14 $$\begin{array}{rl} v_{y}\sin\frac{\theta }{2} & =\sin\frac{\beta }{2}\cos\zeta \end{array}$$

6.15 $$\begin{array}{rl} \text{and}\;v_{z}\sin\frac{\theta }{2} & =\cos\frac{\beta }{2}\sin\eta . \end{array}$$

Hence, it is enough to show that II implies the existence of some η,ζ∈R satisfying (6.12)–(6.15).

Now suppose cos⁡(β/2)≠0. Then, if we show

6.16 $$\frac{\cos^{2}(\theta /2)}{\cos^{2}(\beta /2)}+\frac{v_{z}^{2}\sin^{2}(\theta /2)}{\cos^{2}(\beta /2)}=1,$$

it will immediately imply the existence of *η* satisfying (6.12) and (6.15). From II, however, we have (6.10), and hence, $\left(1-v_{z}^{2})\sin^{2}(\theta /2)=\sin^{2}(\beta /2\right)$, i.e. $1-(1-v_{z}^{2})\sin^{2}(\theta /2)=\cos^{2}(\beta /2)$, which is equivalent to (6.16) by the assumption cos⁡(β/2)≠0. If cos⁡(β/2)=0, then |sin⁡(β/2)|=1. This and (6.10) imply $1-v_{z}^{2}=|\sin(\theta /2)|=1$, and hence, $v_{z}=\cos(\theta /2)=0$. Then, (6.12) and (6.15) hold for any choice of *η*.

In a similar way, if sin⁡(β/2)≠0,

6.17 $$\frac{v_{x}^{2}\sin^{2}(\theta /2)}{\sin^{2}(\beta /2)}+\frac{v_{y}^{2}\sin^{2}(\theta /2)}{\sin^{2}(\beta /2)}=1$$

will immediately imply the existence of *ζ* satisfying (6.13) and (6.14). But (6.17) follows again from II or (6.10) since $1-v_{z}^{2}=v_{x}^{2}+v_{y}^{2}$. If sin⁡(β/2)=0, both (6.13) and (6.14) hold for any choice of *ζ* similarly. ▪

**Proof of proposition 4.4**

Choose a parameter *θ*_*j*_ such that $|\sin(\theta_{j}/2)|=\sin(\beta_{j}/2)/\sin\delta$, which is possible by the assumption *β*_*j*_∈(0,2*δ*]; then, it follows from lemma 6.1 that there exist some $\alpha_{j},\gamma_{j}\in R$ such that (4.5), i.e. $R_{\hat{l}}(\beta_{j})=R_{\hat{m}}(-\alpha_{j})R_{\hat{n}}(\theta_{j})R_{\hat{m}}(-\gamma_{j})$ holds, *j*=1,…,*k*. Inserting these into

$$R_{\hat{m}}(\alpha )R_{\hat{l}}(\beta )R_{\hat{m}}(\gamma )=R_{\hat{m}}(\alpha )R_{\hat{l}}(\beta_{1})\cdots R_{\hat{l}}(\beta_{k})R_{\hat{m}}(\gamma ),$$

we obtain (4.6). ▪

**Proof of proposition 4.7**

**Figure 1.**
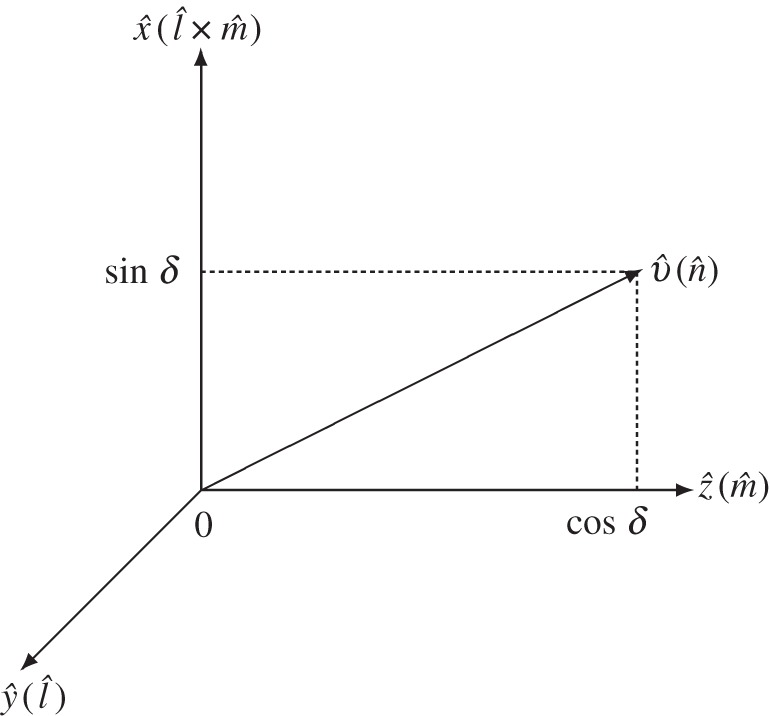
Configuration of $\hat{l},\hat{m}$ and $\hat{n}$ in propositions 4.4 and 4.7, and configuration of $\hat{y}={\left(0,1,0\right)}^{T}$, $\hat{z}={\left(0,0,1\right)}^{T}$ and $\hat{v}$ in arguments around these propositions.

Note $R_{\hat{l}}(\delta )R_{\hat{m}}(\alpha^{\prime })R_{\hat{l}}(-\delta )=R_{\hat{n}}(\alpha^{\prime })$, which is equivalent to *R*_*y*_(*δ*)*R*_*z*_(*α*′)*R*_*y*_(−*δ*)=*R*_*v*_(*α*′), where $\hat{v}={\left(\sin\delta ,0,\cos\delta \right)}^{T}$, by lemma 3.4 (figure 1) and therefore, can be checked easily by a direct calculation. Using this equation, we can rewrite (4.8) as $U=R_{\hat{n}}(\alpha^{\prime })R_{\hat{l}}(\beta^{\prime }+\delta )R_{\hat{m}}(\gamma^{\prime })$, which is (4.9). Then, applying to $R_{\hat{l}}(\beta^{\prime }+\delta )R_{\hat{m}}(\gamma^{\prime })$, the decomposition in proposition 4.4 with (*α*,*β*,*γ*) replaced by (0,*β*′+*δ*,*γ*′), it readily follows that there exist some *α*′_*j*_,*γ*′_*j*_ and $\theta_{j}^{\prime }\in R$, *j*=1,…,*k*′, that satisfy the following: $|\sin(\theta_{j}^{\prime }/2)|=\sin(\beta_{j}^{\prime }/2)/\sin\delta$ and (4.11) for *j*=1,…,*k*′, and

6.18 $$\begin{array}{rl} & R_{\hat{l}}(\beta^{\prime }+\delta )R_{\hat{m}}(\gamma^{\prime })\\ & \;=R_{\hat{m}}(-\alpha_{1}^{\prime })R_{\hat{n}}(\theta_{1}^{\prime })R_{\hat{m}}(-\gamma_{1}^{\prime }-\alpha_{2}^{\prime })R_{\hat{n}}(\theta_{2}^{\prime })R_{\hat{m}}(-\gamma_{2}^{\prime }-\alpha_{3}^{\prime })R_{\hat{n}}(\theta_{3}^{\prime })\cdots \\ & \;\cdot R_{\hat{m}}(-\gamma_{k^{\prime }-1}^{\prime }-\alpha_{k^{\prime }}^{\prime })R_{\hat{n}}(\theta_{k^{\prime }}^{\prime })R_{\hat{m}}(-\gamma_{k^{\prime }}^{\prime }+\gamma^{\prime }). \end{array}$$

Thus, we obtain the proposition. ▪

Remarks 4.6 and 4.9 to these propositions are proved in appendix B. The statement on *α*′_1_ in remark 4.8 follows from remark 4.9 (put *β*′_1_=2*δ* and *t*_1_=*π*/2) or, more directly, from an equation $R_{\hat{l}}(2\delta )=R_{\hat{n}}(\pi )R_{\hat{m}}(-\pi )$, which is equivalent to *R*_*y*_(2*δ*)=*R*_*v*_(*π*)*R*_*z*_(−*π*), where $\hat{v}={\left(\sin\delta ,0,\cos\delta \right)}^{T}$, by lemma 3.4.

### 6.3 Proof of theorem 4.3

Let 2N−1 and 2N denote the set of odd numbers in N and that of even numbers in N, respectively. We define the following for $\hat{m},\hat{n}\in S^{2}$ with $|{\hat{m}}^{T}\hat{n}|<1$:

$$\begin{array}{rl} M_{\hat{m},\hat{n}}^{\mathrm{odd}}(U):=\min\{j\in 2N-1| & \exists V_{1},V_{3},\ldots ,V_{j}\in R_{\hat{m}},\\ & \exists V_{2},V_{4},\ldots ,V_{j-1}\in R_{\hat{n}},\;U=V_{j}V_{j-1}\cdots V_{1}\},\\ M_{\hat{m},\hat{n}}^{\mathrm{even}}(U):=\min\{j\in 2N| & \exists V_{1},V_{3},\ldots ,V_{j-1}\in R_{\hat{m}},\\ & \exists V_{2},V_{4},\ldots ,V_{j}\in R_{\hat{n}},\;U=V_{j}V_{j-1}\cdots V_{1}\}\\ \mathrm{and}\;M_{\hat{m},\hat{n}}(U) & :=\min\{M_{\hat{m},\hat{n}}^{\mathrm{odd}}(U),\;M_{\hat{m},\hat{n}}^{\mathrm{even}}(U)\} \end{array}$$

for *U*∈SU(2);

$$\begin{array}{rl} M_{\hat{m},\hat{n}}^{\mathrm{odd}}(D):=\min\{j\in 2N-1| & \exists A_{1},A_{3},\ldots ,A_{j}\in {\hat{R}}_{\hat{m}},\\ & \exists A_{2},A_{4},\ldots ,A_{j-1}\in {\hat{R}}_{\hat{n}},\;D=A_{j}A_{j-1}\cdots A_{1}\},\\ M_{\hat{m},\hat{n}}^{\mathrm{even}}(D):=\min\{j\in 2N| & \exists A_{1},A_{3},\ldots ,A_{j-1}\in {\hat{R}}_{\hat{m}},\\ & \exists A_{2},A_{4},\ldots ,A_{j}\in {\hat{R}}_{\hat{n}},\;D=A_{j}A_{j-1}\cdots A_{1}\},\\ \mathrm{and}\;M_{\hat{m},\hat{n}}(D) & :=\min\{M_{\hat{m},\hat{n}}^{\mathrm{odd}}(D),\;M_{\hat{m},\hat{n}}^{\mathrm{even}}(D)\} \end{array}$$

for *D*∈SO(3). The following lemma largely solves the issue of determining the optimal number $N_{\hat{m},\hat{n}}(U)$.

**Lemma 6.2**

*Let* $\hat{m},\hat{n},$ $\hat{l}$ *and* *δ* *be as in theorem* 4.3. *Then, for any* α,γ∈R *and* *β*∈[0,*π*],

6.19 $$M_{\hat{m},\hat{n}}^{\mathrm{odd}}(F(U_{\alpha ,\beta ,\gamma }^{\hat{m},\hat{l}}))=M_{\hat{m},\hat{n}}^{\mathrm{odd}}(U_{\alpha ,\beta ,\gamma }^{\hat{m},\hat{l}})=2\left\lceil \frac{\beta }{2\delta }\right\rceil +1$$

*and*

6.20 $$M_{\hat{m},\hat{n}}^{\mathrm{even}}(F(U_{\alpha ,\beta ,\gamma }^{\hat{m},\hat{l}}))=M_{\hat{m},\hat{n}}^{\mathrm{even}}(U_{\alpha ,\beta ,\gamma }^{\hat{m},\hat{l}})=g(\alpha ,\beta ,\delta ),$$

*where* $U_{\alpha ,\beta ,\gamma }^{\hat{m},\hat{l}}$ *is as defined in theorem* 4.3.

**Corollary 6.3**

*Let* $\hat{m},\hat{n},$ $\hat{l}$ *and* *δ* *be as in theorem* 4.3. *Then, for any* α,γ∈R *and* *β*∈[0,*π*],

6.21 $$M_{\hat{m},\hat{n}}(F(U_{\alpha ,\beta ,\gamma }^{\hat{m},\hat{l}}))=M_{\hat{m},\hat{n}}(U_{\alpha ,\beta ,\gamma }^{\hat{m},\hat{l}})=\min\left\{2\left\lceil \frac{\beta }{2\delta }\right\rceil +1,g(\alpha ,\beta ,\delta )\right\}.$$

**Proof**

In the case where *β*=0, since $M_{\hat{m},\hat{n}}^{\mathrm{odd}}(U_{\alpha ,\beta ,\gamma }^{\hat{m},\hat{l}})=1$ and $M_{\hat{m},\hat{n}}^{\mathrm{even}}(U_{\alpha ,\beta ,\gamma }^{\hat{m},\hat{l}})=2$, (6.19) and (6.20) are trivially true. We shall prove the statement for *β*>0.

To establish (6.19), we shall show the first and third inequalities in

6.22 $$2\left\lceil \frac{\beta }{2\delta }\right\rceil +1\leq M_{\hat{m},\hat{n}}^{\mathrm{odd}}(F(U_{\alpha ,\beta ,\gamma }^{\hat{m},\hat{l}}))\leq M_{\hat{m},\hat{n}}^{\mathrm{odd}}(U_{\alpha ,\beta ,\gamma }^{\hat{m},\hat{l}})\leq 2\left\lceil \frac{\beta }{2\delta }\right\rceil +1$$

while the second inequality trivially follows from the definition of $M_{\hat{m},\hat{n}}^{\mathrm{odd}}$.

Note first that remark 4.5 to proposition 4.4 immediately implies the third inequality in (6.22). To prove the first inequality, assume

6.23 $$F(U_{\alpha ,\beta ,\gamma }^{\hat{m},\hat{l}})=A_{j}A_{j-1}\cdots A_{1}$$

for some *j*=2*k*−1 with k∈N, where $A_{\nu }\in {\hat{R}}_{\hat{m}}$ if *ν* is odd and $A_{\nu }\in {\hat{R}}_{\hat{n}}$ otherwise.

We shall evaluate $d(F(U_{\alpha ,\beta ,\gamma }^{\hat{m},\hat{l}})\hat{m},\hat{m})=d(A_{j}A_{j-1}\cdots A_{1}\hat{m},\hat{m})$. Noting that $d(F(U_{\alpha ,\beta ,\gamma }^{\hat{m},\hat{l}})\hat{m},\hat{m})=\beta$, we have *β*≤2(*k*−1)*δ* by (5.2) of lemma 4.1. This implies ⌈*β*/(2*δ*)⌉≤*k*−1, and therefore,

6.24 $$2\left\lceil \frac{\beta }{2\delta }\right\rceil +1\leq 2k-1=j.$$

From this bound, we have the first inequality in (6.22), and hence (6.19).

To establish (6.20), we shall first treat the major case where *f*(*α*,*β*,*δ*)≥*δ*. Recalling that $g(\alpha ,\beta ,\delta )=2\lceil f(\alpha ,\beta ,\delta )/(2\delta )+\frac{1}{2}\rceil$ in this case, we shall show the first and third inequalities in

6.25 $$2\left\lceil \frac{f(\alpha ,\beta ,\delta )}{2\delta }+\frac{1}{2}\right\rceil \leq M_{\hat{m},\hat{n}}^{\mathrm{even}}(F(U_{\alpha ,\beta ,\gamma }^{\hat{m},\hat{l}}))\leq M_{\hat{m},\hat{n}}^{\mathrm{even}}(U_{\alpha ,\beta ,\gamma }^{\hat{m},\hat{l}})\leq 2\left\lceil \frac{f(\alpha ,\beta ,\delta )}{2\delta }+\frac{1}{2}\right\rceil$$

while the second inequality holds trivially.

Note that remark 4.8 to proposition 4.7 will imply the third inequality upon showing that *β*′ in proposition 4.7 satisfies *β*′=*f*(*α*,*β*,*δ*) when $U=U_{\alpha ,\beta ,\gamma }^{\hat{m},\hat{l}}$. To see *β*′=*f*(*α*,*β*,*δ*), rewrite (4.8), using lemma 3.4, as

6.26 $$R_{y}(-\delta )R_{z}(\alpha )R_{y}(\beta )R_{z}(\gamma )=R_{z}(\alpha^{\prime })R_{y}(\beta^{\prime })R_{z}(\gamma^{\prime }).$$

Then, a direct calculation shows the absolute value of the (1,1)-entry of the left-hand side equals

$$\sqrt{\cos^{2}\frac{\beta }{2}\cos^{2}\frac{\delta }{2}+\sin^{2}\frac{\beta }{2}\sin^{2}\frac{\delta }{2}+2\cos\alpha \sin\frac{\beta }{2}\sin\frac{\delta }{2}\cos\frac{\beta }{2}\cos\frac{\delta }{2}}.$$

This shows *β*′=*f*(*α*,*β*,*δ*) in view of (3.6).

To prove the first inequality in (6.25), assume (6.23) holds for some *j*=2*k* with k∈N, where $A_{\nu }\in {\hat{R}}_{\hat{m}}$ if *ν* is odd and $A_{\nu }\in {\hat{R}}_{\hat{n}}$ otherwise. Note that $\hat{n}={\hat{R}}_{\hat{l}}(\delta )\hat{m}$ and hence, for $U=R_{\hat{n}}(\alpha^{\prime })R_{\hat{l}}(\beta^{\prime }+\delta )R_{\hat{m}}(\gamma^{\prime })$ in proposition 3.7,

$$d(F(U)\hat{m},\hat{n})=d({\hat{R}}_{\hat{l}}(\beta^{\prime }+\delta )\hat{m},\hat{n})=d({\hat{R}}_{\hat{l}}(\beta^{\prime }+\delta )\hat{m},{\hat{R}}_{\hat{l}}(\delta )\hat{m})=(\beta^{\prime }+\delta )-\delta =\beta^{\prime }.$$

Then, we have *β*′≤(2*k*−1)*δ* by (5.4) of lemma 5.1. This implies ⌈(*β*′+*δ*)/(2*δ*)⌉≤*k*, and, therefore,

6.27 $$2\left\lceil \frac{\beta^{\prime }+\delta }{2\delta }\right\rceil \leq 2k=j.$$

From this bound, we have the first inequality in (6.25) and, hence, the equality among all sides of (6.25). This shows (6.20) in the case where *f*(*α*,*β*,*δ*)≥*δ*. The proof of (6.20) in the other case is given in appendix C. This completes the proof of the lemma. The proved lemma immediately implies the corollary. ▪

**Proof of theorem 4.3**

Note that for any *U*∈SU(2),

$$N_{\hat{m},\hat{n}}(U)=\min\{M_{\hat{m},\hat{n}}^{\mathrm{odd}}(U),M_{\hat{m},\hat{n}}^{\mathrm{even}}(U),M_{\hat{n},\hat{m}}^{\mathrm{odd}}(U),M_{\hat{n},\hat{m}}^{\mathrm{even}}(U)\},$$

and we can write *U* in terms of three parametric expressions:

$$U=R_{\hat{u}}(\theta )=U_{\alpha ,\beta ,\gamma }^{\hat{m},\hat{l}}=U_{\tilde{\alpha },\tilde{\beta },\tilde{\gamma }}^{\hat{n},-\hat{l}},$$

where $\beta ,\tilde{\beta }\in [0,\pi ]$, $\alpha ,\gamma ,\tilde{\alpha },\tilde{\gamma },\theta \in R$ and $\hat{u}\in S^{2}$. Then, we have

$$\frac{\beta }{2}=\arcsin\left[\sqrt{1-{\left({\hat{m}}^{T}\hat{u}\right)}^{2}}\left|\sin\frac{\theta }{2}\right|\right]\;\text{and}\;\frac{\tilde{\beta }}{2}=\arcsin\left[\sqrt{1-{\left({\hat{n}}^{T}\hat{u}\right)}^{2}}\left|\sin\frac{\theta }{2}\right|\right]$$

owing to lemma 5.1, and, hence,

$$M_{\hat{m},\hat{n}}^{\mathrm{odd}}(U)=2\left\lceil \frac{\arcsin\sqrt{1-{\left({\hat{m}}^{T}\hat{u}\right)}^{2}}|\sin(\theta /2)|}{\delta }\right\rceil +1$$

and

$$M_{\hat{n},\hat{m}}^{\mathrm{odd}}(U)=2\left\lceil \frac{\arcsin\sqrt{1-{\left({\hat{n}}^{T}\hat{u}\right)}^{2}}|\sin(\theta /2)|}{\delta }\right\rceil +1$$

owing to lemma 5.2. Then, if $|{\hat{m}}^{T}\hat{u}|\geq |{\hat{n}}^{T}\hat{u}|$ whenever sin⁡(θ/2)≠0, which implies $M_{\hat{m},\hat{n}}^{\mathrm{odd}}(U)\leq M_{\hat{n},\hat{m}}^{\mathrm{odd}}(U)$, we shall have

6.28 $$\begin{array}{rl} N_{\hat{m},\hat{n}}(U) & =\min\{M_{\hat{m},\hat{n}}^{\mathrm{odd}}(U),M_{\hat{m},\hat{n}}^{\mathrm{even}}(U),M_{\hat{n},\hat{m}}^{\mathrm{even}}(U)\}\\ & =\min\left\{2\left\lceil \frac{\beta }{2\delta }\right\rceil +1,g(\alpha ,\beta ,\delta ),M_{\hat{n},\hat{m}}^{\mathrm{even}}(U)\right\} \end{array}$$

for $U=U_{\alpha ,\beta ,\gamma }^{\hat{m},\hat{l}}$. But $\left[\sin(\theta /2)\neq 0\to |{\hat{m}}^{T}\hat{u}|\geq |{\hat{n}}^{T}\hat{u}|\right]$ follows from $b(\hat{m},U_{\alpha ,\beta ,\gamma }^{\hat{m},\hat{l}})\geq b(\hat{n},U_{\alpha ,\beta ,\gamma }^{\hat{m},\hat{l}})$ by the definition of *b*. (This is because writing *U* in (4.1) as $U=R_{\hat{u}}(\theta )$, θ∈R, $\hat{u}\in S^{2}$, results in $-\sin(\theta /2)\hat{u}={\left(x,y,z\right)}^{T}$ as in §2.3, whereby $b(\hat{v},U)=|\sin(\theta /2)||{\hat{u}}^{T}\hat{v}|$.) Hence, we have (6.28).

A short additional argument (appendix D) shows

6.29 $$M_{\hat{n},\hat{m}}^{\mathrm{even}}(U_{\alpha ,\beta ,\gamma }^{\hat{m},\hat{l}})=g(\gamma ,-\beta ,\delta ),$$

and, therefore,

$$N_{\hat{m},\hat{n}}(U_{\alpha ,\beta ,\gamma }^{\hat{m},\hat{l}})=\min\left\{2\left\lceil \frac{\beta }{2\delta }\right\rceil +1,g(\alpha ,\beta ,\delta ),g(\gamma ,-\beta ,\delta )\right\}.$$

Finally, from corollary 6.3 or from the argument in appendix E, it readily follows that $N_{\hat{m},\hat{n}}(F(U_{\alpha ,\beta ,\gamma }^{\hat{m},\hat{l}}))=N_{\hat{m},\hat{n}}(U_{\alpha ,\beta ,\gamma }^{\hat{m},\hat{l}})$. Hence, we obtain the theorem. ▪

From the viewpoint of construction, we summarize the (most directly) suggested way to obtain an optimal construction of a given element *U*∈SU(2), where we assume $\delta =\arccos{\hat{m}}^{T}\hat{n}\in (0,\pi /2]$ without loss of generality. If $b(\hat{m},U)$ $\geq b(\hat{n},U)$, choose a construction that attains the minimum in (6.28). The construction is among that of proposition 4.4, that of proposition 4.7 and that of proposition 4.7 applied to *U*^†^ in place of *U* [note $U^{†}=R_{{\hat{u}}_{1}}(\phi_{1})\cdots R_{{\hat{u}}_{j}}(\phi_{j})$ implies $U=R_{{\hat{u}}_{j}}(-\phi_{j})\cdots R_{{\hat{u}}_{1}}(-\phi_{1})$]. If $b(\hat{m},U)<b(\hat{n},U)$, interchanging $\hat{m}$ and $\hat{n}$, apply the construction just described.^7^ See appendix G for a detailed description of the above construction method.

## 7. Conclusion

This work has established the least value $N_{\hat{m},\hat{n}}(U)$ of a positive integer *k* such that *U* can be decomposed into the product of *k* rotations about either $\hat{m}$ or $\hat{n}$ for an arbitrarily fixed element *U* in SU(2), or in SO(3), where $\hat{m},\hat{n}\in S^{2}$ are arbitrary real unit vectors with $|{\hat{m}}^{T}\hat{n}|<1$. Decompositions of *U* attaining the minimum number $N_{\hat{m},\hat{n}}(U)$ have also been given explicitly.

## 8. Comments on Brezov *et al*. [10–12]

In this paper, an algorithm for solving the following unusual optimization problem was presented:

$$\begin{array}{rl} \text{minimize}\; & \mathrm{length}(\tau_{1},\ldots ,\tau_{\nu },{\hat{m}}_{1},\ldots ,{\hat{m}}_{\nu })\\ \text{subject to}\; & R_{{\hat{m}}_{1}}(\tau_{1})R_{{\hat{m}}_{2}}(\tau_{2})\cdots R_{{\hat{m}}_{\nu }}(\tau_{\nu })=U,\\ & \nu \in N;\;\tau_{j}\in R,{\hat{m}}_{j}\in A\;\text{for }j=1,\ldots ,\nu \end{array}$$

where $\mathrm{length}(\tau_{1},\ldots ,\tau_{\nu },{\hat{m}}_{1},\ldots ,{\hat{m}}_{\nu }):=\nu$, *U* is an arbitrary fixed rotation and *A*⊂*S*^2^ with |*A*|=2 (the minimum of ‘length’, the primary part of an optimal solution, has been denoted by $N_{\hat{m},\hat{n}}(U)$). To this author's knowledge, only the work by D'Alessandro [5] and this paper have discussed this optimization problem.

Naturally, the present author could not find any (explicit or implicit) indication that Brezov *et al*. [10–12] suggest considering the quantity $N_{\hat{m},\hat{n}}(U)$ or analogues. A difference in background between this paper and Brezov *et al*. [10–12] may be understood as follows. While the situation assumed in this paper is that only two axes are available in constructing an arbitrary rotation, assuming a different situation results in problem formulations different from ours. For example, in Leite [7, Lemma 4.2] (attributed to Davenport), a situation where three axes are available but the number of factors in a decomposition is limited to three or less (in words, an equation $R_{{\hat{m}}_{1}}(\tau_{1})R_{{\hat{m}}_{2}}(\tau_{2})R_{{\hat{m}}_{3}}(\tau_{3})=U$, i.e. the above equation with *ν*=3) is considered. In the series of Brezov *et al*. [10–12], they investigated such decompositions of the Davenport type, seemingly with emphasis on physical aspects. Note that $N_{\hat{m},\hat{n}}(\mathrm{SU}(2))=\max_{U}N_{\hat{m},\hat{n}}(U)=\lceil \pi /\arccos|{\hat{m}}^{T}\hat{n}|\rceil +1$, $\hat{m}\neq \pm \hat{n}$, is greater than three except in the classical case, where $\hat{m}$ and $\hat{n}$ are orthogonal to each other.

Despite such differences in essence and background, note in the proof of this paper's formula (6.20) for the minimum even number of factors in lemma 6.2, on which the main theorem (theorem 4.3) relies, the case where the minimum even number is 2 or 4 needs an exceptional treatment (appendix C). This exceptionality would motivate one to read treatments on decompositions into two factors, and such can be found in Brezov *et al*. [10–12].

## Footnotes

1 Here, the crux of the difficulty in obtaining this work's results will be explained. Finding the minimum *odd* number of factors needed for decomposing *U*, which is expressed with a standard parameter *β* of *U*, together with minimum-achieving decompositions, was relatively easy. The crux lay in obtaining a solution to attain the minimum *even* number of factors, which was found to be expressed with another parameter *β*′ eventually.

2 The restriction of *β* to [0,*π*] does not seem common. However, in a straightforward proof of this lemma, *β*∈[0,*π*] can be chosen so that cos⁡(β/2)=|a| and sin⁡(β/2)=|b| when the first row of *U* is (*a*,*b*). Also any *R*_*z*_(*α*′)*R*_*y*_(*β*′)*R*_*z*_(*γ*′) without this restriction can be written as *R*_*z*_(*α*)*R*_*y*_(*β*)*R*_*z*_(*γ*) with some *β*∈[0,*π*] and α,γ∈R. This readily follows from equations $R_{\hat{v}}(\theta +2\pi )=-R_{\hat{v}}(\theta )$, $\hat{v}\in S^{2}$, θ∈R, and *R*_*z*_(−*π*)*R*_*y*_(*β*′)*R*_*z*_(*π*)=*R*_*y*_(−*β*′), $\beta^{\prime }\in R$.

3 The objects treated in this subsection and previous one can be found in Wigner [3, ch. 15], where −*Y* and −*Z* have been used instead of our *Y* and *Z* in defining the homomorphism. Owing to this difference, the homomorphism in Wigner [3] is *TF*(*U*)*T*, where *T* is the diagonal matrix with diagonal entries 1 (leftmost), −1 and −1. (For example, *TF*(*R*_*y*_(*θ*))*T* and *TF*(*R*_*z*_(*θ*))*T* have appeared in Wigner [3, ch. 15] while we shall use *F*(*R*_*y*_(*θ*)) and *F*(*R*_*z*_(*θ*)) in appendix A. The general form $R_{\hat{v}}(\theta )$ can be derived in a natural manner, but one may consult Biedenharn & Louck [4, ch. 2] for it.)

4 For the sake of constructiveness, such an element *U* is constructed in appendix A.

5 To make the construction explicit, one can set *β*_*j*_=2*δ* for *j*≠*k*. The analogous comment applies to the division of *β*′+*δ* in proposition 4.7.

6 All remarks except remark 4.5, which needs no proof, will be proved in what follows.

7 One (seemingly difficult) issue arises: determine all optimal decompositions of an arbitrarily fixed rotation. Note that in propositions 4.4 and 4.7 and their proofs, any solution for $R_{\hat{n}}(\theta )=R_{\hat{m}}(\alpha )R_{\hat{l}}(\beta )R_{\hat{m}}(\gamma )$ can be used (see corollary B.2 in appendix B for explicit solutions, among which one is chosen to be used in remarks 4.6 and 4.9).

## Appendix A. Element in SU(2) associated with $\hat{l}$ and $\hat{m}$

Our goal here is to prove (in a constructive manner) that for any pair of vectors $\hat{l},\hat{m}\in S^{2}$ with $\hat{l}{\;}^{T}\hat{m}=0$, there exists some element *U*∈SU(2) such that $\hat{l}=F(U){\left(0,1,0\right)}^{T}$ and $\hat{m}=F(U){\left(0,0,1\right)}^{T}$. Expressing *U* as $U=R_{z}(\tilde{\alpha })R_{y}(\tilde{\beta })R_{z}(\tilde{\gamma })$, we shall specify desired $\tilde{\alpha },\tilde{\beta }$ and $\tilde{\gamma }$. By a direct calculation with

$${\hat{R}}_{y}(\theta )=\left(\begin{array}{ccc} \cos\theta & 0 & \sin\theta \\ 0 & 1 & 0\\ -\sin\theta & 0 & \cos\theta \end{array}\right)\;\text{and}\;{\hat{R}}_{z}(\theta )=\left(\begin{array}{ccc} \cos\theta & -\sin\theta & 0\\ \sin\theta & \cos\theta & 0\\ 0 & 0 & 1 \end{array}\right),$$

where ${\hat{R}}_{y}(\theta ):=F(R_{y}(\theta ))$ and ${\hat{R}}_{z}(\theta ):=F(R_{z}(\theta ))$, we have $F(U){\left(0,0,1\right)}^{T}={\left(\cos\tilde{\alpha }\sin\tilde{\beta },\sin\tilde{\alpha }\sin\tilde{\beta },\cos\tilde{\beta }\right)}^{T}$. On the other hand, the condition $\hat{l}=F(U){\left(0,1,0\right)}^{T}$ is equivalent to ${\hat{R}}_{y}(-\tilde{\beta }){\hat{R}}_{z}(-\tilde{\alpha })\hat{l}={\hat{R}}_{z}(\tilde{\gamma }){\left(0,1,0\right)}^{T}$, i.e.

A 1 $$\left(\begin{array}{ccc} \cos\tilde{\beta }\cos\tilde{\alpha } & \cos\tilde{\beta }\sin\tilde{\alpha } & -\sin\tilde{\beta }\\ -\sin\tilde{\alpha } & \cos\tilde{\alpha } & 0\\ \cos\tilde{\alpha }\sin\tilde{\beta } & \sin\tilde{\alpha }\sin\tilde{\beta } & \cos\tilde{\beta } \end{array}\right)\hat{l}=\left(\begin{array}{c} -\sin\tilde{\gamma }\\ \cos\tilde{\gamma }\\ 0 \end{array}\right).$$

Hence, choosing parameters $\tilde{\alpha }$ and $\tilde{\beta }$ such that ${\left(\cos\tilde{\alpha }\sin\tilde{\beta },\sin\tilde{\alpha }\sin\tilde{\beta },\cos\tilde{\beta }\right)}^{T}=\hat{m}$, cf. spherical coordinates, and $\tilde{\gamma }$ that satisfies (A.1), we have a desired element $U=R_{z}(\tilde{\alpha })R_{y}(\tilde{\beta })R_{z}(\tilde{\gamma })$ such that $\hat{l}=F(U){\left(0,1,0\right)}^{T}$ and $\hat{m}=F(U){\left(0,0,1\right)}^{T}$.

## Appendix B. Details on angles in propositions 4.4 and 4.7

Examining the proof of lemma 6.1, we can be specific about *α* and *γ* to have the following lemma and corollary. In particular, the corollary gives a sufficient condition, (i), and two necessary conditions, (ii) and (iii), for $R_{\hat{n}}(\theta )=R_{\hat{m}}(\alpha )R_{\hat{l}}(\beta )R_{\hat{m}}(\gamma )$, where $\hat{l},\hat{m}$ and $\hat{n}$ are set as in propositions 4.4 and 4.7. Remarks 4.6 and 4.9 will be clear from (i). Later, (ii) and (iii) will be used in appendices C and D, respectively, though the use of them is not mandatory.

Lemma B.1 *For any* θ,α,β,γ∈R, *and* $\hat{n},\hat{l},\hat{m}\in S^{2}$ *such that* $\hat{l}{\;}^{T}\hat{m}=0,$

B 1 $$R_{\hat{n}}(\theta )=R_{\hat{m}}(\alpha )R_{\hat{l}}(\beta )R_{\hat{m}}(\gamma )$$

*holds iff the following conditions hold*:

B 2 $$\cos\frac{\gamma +\alpha }{2}=\frac{\cos(\theta /2)}{\cos(\beta /2)}\;\text{and}\;\sin\frac{\gamma +\alpha }{2}=\frac{{\hat{m}}^{T}\hat{n}\sin(\theta /2)}{\cos(\beta /2)}$$

*whenever* cos⁡(β/2)≠0,

B 3 $$\sin\frac{\gamma -\alpha }{2}=\frac{{\left(\hat{l}\times \hat{m}\right)}^{T}\hat{n}\sin(\theta /2)}{\sin(\beta /2)}\;\text{and}\;\cos\frac{\gamma -\alpha }{2}=\frac{\hat{l}{\;}^{T}\hat{n}\sin(\theta /2)}{\sin(\beta /2)}$$

*whenever* sin⁡(β/2)≠0, *and*

B 4 $$\sqrt{1-{\left({\hat{m}}^{T}\hat{n}\right)}^{2}}\left|\sin\frac{\theta }{2}\right|=\left|\sin\frac{\beta }{2}\right|.$$

Corollary B.2 *Given any* *δ*∈(0,*π*/2] *and* $\hat{l},\hat{m}\in S^{2}$ *such that* $\hat{l}{\;}^{T}\hat{m}=0,$ *put*

B 5 $$\hat{n}=(\sin\delta )\hat{l}\times \hat{m}+(\cos\delta )\hat{m}.$$

*Then*, (*i*) *for any* θ,α,γ∈R *and* *β*∈[0,*π*], (B 1) *holds if*

β≤2δ

*and there exists some* t∈R *such that* (*recall* *H*_*t*_ *is defined in remark* 4.6)

B 6 $$\left(\begin{array}{c} \alpha \\ \gamma \\ \theta \end{array}\right)=\pm \left(\begin{array}{c} H_{t}(\beta ,\delta )-\frac{\pi }{2}\\ H_{t}(\beta ,\delta )+\frac{\pi }{2}\\ \;2\arcsin\frac{\sin(\beta /2)}{\sin\delta } \end{array}\right)\;\text{or}\;\left(\begin{array}{c} \alpha \\ \gamma \\ \theta \end{array}\right)=\pm \left(\begin{array}{c} -H_{t}(\beta ,\delta )+\frac{\pi }{2}\\ -H_{t}(\beta ,\delta )+\frac{3\pi }{2}\\ \;2\pi -2\arcsin\frac{\sin(\beta /2)}{\sin\delta } \end{array}\right);$$

(ii) *for any* α∈R *and* *β*∈(0,*π*], *if* (B 1) *holds for some* θ,γ∈R, *then* *β*≤2*δ* *and there exist some* j∈Z *and* t∈R *such that*^8^

$$\alpha =\pm H_{t}(\beta ,\delta )\pm \frac{\pi }{2}+\pi j;$$

(iii) *for any* γ∈R *and* *β*∈(0,*π*], *if* (B 1) *holds for some* θ,α∈R, *then* *β*≤2*δ* *and there exist some* j∈Z *and* t∈R *such that*

$$\gamma =\pm H_{t}(\beta ,\delta )\pm \frac{\pi }{2}+\pi j.$$

Proof Set $\hat{v}={\left(v_{x},v_{y},v_{z}\right)}^{T}$ with

$$v_{x}={\left(\hat{l}\times \hat{m}\right)}^{T}\hat{n},\;v_{y}=\hat{l}{\;}^{T}\hat{n}\;\text{and}\;v_{z}={\hat{m}}^{T}\hat{n}.$$

Then, according to paragraphs (1) and (2) in the proof of lemma 6.1, for any θ,α,β,γ∈R, (B 1) holds iff (6.6)–(6.9) hold. But (6.6)–(6.9) hold iff (B 4), [cos⁡(β/2)≠0→] and [sin⁡(β/2)≠0→] hold. This completes the proof of the lemma. To see the corollary (recall figure 1 and), note

$${\left(\hat{l}\times \hat{m}\right)}^{T}\hat{n}=\sin\delta ,\;\hat{l}{\;}^{T}\hat{n}=0\;\text{and}\;{\hat{m}}^{T}\hat{n}=\cos\delta .$$

Then, (B 4), [cos⁡(β/2)≠0→] and [sin⁡(β/2)≠0→] hold if the following two conditions are satisfied: (a) *β*≤2*δ* and (b)

$$\left\{ \begin{array}{l} \frac{\gamma +\alpha }{2}=\arcsin\frac{\tan(\beta /2)}{\tan\delta }\\ \frac{\gamma -\alpha }{2}=\frac{\pi }{2}\\ \theta =2\arcsin\frac{\sin(\beta /2)}{\sin\delta } \end{array}\right.\;\text{or}\;\left\{ \begin{array}{l} \frac{\gamma +\alpha }{2}=\pi -\arcsin\frac{\tan(\beta /2)}{\tan\delta }\\ \frac{\gamma -\alpha }{2}=\frac{\pi }{2}\\ \theta =2\pi -2\arcsin\frac{\sin(\beta /2)}{\sin\delta } \end{array}\right.$$

unless *β*/2=*δ*=*π*/2,^9^ and

$$\left\{ \begin{array}{rl} & \frac{\gamma +\alpha }{2}=s\\ & \frac{\gamma -\alpha }{2}=\frac{\pi }{2}\\ & \theta =\beta \end{array}\right.$$

for some s∈R if *β*/2=*δ*=*π*/2. This readily gives two solutions for (B 1). Rewriting these solutions with *H*_*t*_ and checking that flipping the signs of the solutions gives other solutions, we obtain (i). Showing (ii) and (iii) is as easy as showing (i). ▪

## Appendix C. Proofs of (6.20) in the case *f*(*α*,*β*,*δ*)<*δ*

Proof 1 Proposition 4.7 and remark 4.8 show $M_{\hat{m},\hat{n}}^{\mathrm{even}}(U_{\alpha ,\beta ,\gamma }^{\hat{m},\hat{l}})\leq 4$, i.e. either $M_{\hat{m},\hat{n}}^{\mathrm{even}}(U_{\alpha ,\beta ,\gamma }^{\hat{m},\hat{l}})=2$ or $M_{\hat{m},\hat{n}}^{\mathrm{even}}(U_{\alpha ,\beta ,\gamma }^{\hat{m},\hat{l}})=4$. We also have $M_{\hat{m},\hat{n}}^{\mathrm{even}}(F(U))=M_{\hat{m},\hat{n}}^{\mathrm{even}}(U)$ for any *U*∈SU(2) (appendix E). Hence, all we need to show is that

C 1 $$\exists \theta ,\;\phi \in R,\;R_{\hat{m}}(\alpha )R_{\hat{l}}(\beta )R_{\hat{m}}(\gamma )=R_{\hat{n}}(\theta )R_{\hat{m}}(\phi )$$

implies *f*(*α*,*β*,*δ*)≥*δ*. This can be shown easily with corollary B.2, (ii). ▪

Proof 2 We shall show that (C 1), i.e.

C 2 $$\exists \theta ,\;\tilde{\gamma }\in R,\;R_{\hat{m}}(\alpha )R_{\hat{l}}(\beta )R_{\hat{m}}(\tilde{\gamma })=R_{\hat{n}}(\theta ),$$

implies *f*(*α*,*β*,*δ*)=*δ*, which is enough. Note that *f*(*α*,*β*,*δ*)=*β*′ for the angle *β*′∈[0,*π*] such that

C 3 $$\exists \alpha^{\prime },\;\gamma^{\prime }\in R,\;R_{\hat{l}}(-\delta )R_{\hat{m}}(\alpha )R_{\hat{l}}(\beta )R_{\hat{m}}(\gamma )=R_{\hat{m}}(\alpha^{\prime })R_{\hat{l}}(\beta^{\prime })R_{\hat{m}}(\gamma^{\prime })$$

(Proof of lemma 6.2 in §6.3). From (C 2) and (C 3), we have

$$\exists \alpha^{\prime },\;\gamma^{\prime },\;\tilde{\gamma },\;\theta \in R,\;R_{\hat{m}}(\alpha^{\prime })R_{\hat{l}}(\beta^{\prime })R_{\hat{m}}(\gamma^{\prime }-\gamma +\tilde{\gamma })=R_{\hat{l}}(-\delta )R_{\hat{n}}(\theta ),$$

which is, by lemma 3.4, equivalent to

C 4 $$\exists \alpha^{\prime },\;\gamma^{\prime },\;\tilde{\gamma },\;\theta \in R,\;R_{z}(\alpha^{\prime })R_{y}(\beta^{\prime })R_{z}(\gamma^{\prime }-\gamma +\tilde{\gamma })=R_{y}(-\delta )R_{\hat{v}}(\theta ),$$

where $\hat{v}={\left(\sin\delta ,0,\cos\delta \right)}^{T}$. The absolute value of the (1,1)-entry of the right-hand side in (C 4) equals cos⁡(δ/2) since *R*_*y*_(−*δ*)*R*_*v*_(*θ*)=*R*_*z*_(*θ*)*R*_*y*_(−*δ*), which is equivalent to the equation *R*_*v*_(*θ*)=*R*_*y*_(*δ*)*R*_*z*_(*θ*)*R*_*y*_(−*δ*) used before. In view of (3.6), this implies *β*′=*δ*, i.e. *f*(*α*,*β*,*δ*)=*δ* as desired. ▪

## Appendix D. Proof of (6.29)

Observe that $M_{\hat{n},\hat{m}}^{\mathrm{even}}(U)=M_{\hat{m},\hat{n}}^{\mathrm{even}}(U^{†})$ for any *U*∈SU(2), by definition, and also that ${\left(U_{\alpha ,\beta ,\gamma }^{\hat{m},\hat{l}}\right)}^{†}=U_{-\gamma ,-\beta ,-\alpha }^{\hat{m},\hat{l}}=U_{-\gamma -\pi ,\beta ,-\alpha +\pi }^{\hat{m},\hat{l}}$ for any *α*,*γ* and *β*∈[0,*π*], cf. footnote 2. These facts give $M_{\hat{n},\hat{m}}^{\mathrm{even}}(U_{\alpha ,\beta ,\gamma }^{\hat{m},\hat{l}})=g(-\gamma -\pi ,\beta ,\delta )=g(\gamma ,-\beta ,\delta )$ as desired.^10^

## Appendix E. Proof that $M_{\hat{m},\hat{n}}^{\mathrm{even}}(F(U))=M_{\hat{m},\hat{n}}^{\mathrm{even}}(U)$ and $N_{\hat{m},\hat{n}}(F(U))=N_{\hat{m},\hat{n}}(U)$

Let any $\hat{m},\hat{n}\in S^{2}$ with $|{\hat{m}}^{T}\hat{n}|<1$ and *U*∈SU(2) be given. By definition, $M_{\hat{m},\hat{n}}^{\mathrm{even}}(F(U))\leq M_{\hat{m},\hat{n}}^{\mathrm{even}}(U)$. We shall show the inequality in the other direction using the following lemma.

Lemma E.1 *For any* *U*,*V* ∈SU(2), *F*(*U*)=*F*(*V*) *iff* *U*=±*V* .

Proof This directly follows from the well-known fact that the kernel of *F* is {*I*,−*I*}, which can be checked with (3.1). ▪

From this lemma, it readily follows that if there exist some j∈N, ${\hat{v}}_{1},\ldots ,{\hat{v}}_{j}\in S^{2}$ and *ϕ*_1_,…,*ϕ*_*j*_ ∈R such that $F(U)=F(R_{{\hat{v}}_{1}}(\phi_{1}))\cdots F(R_{{\hat{v}}_{j}}(\phi_{j}))$, then $U=\pm R_{{\hat{v}}_{1}}(\phi_{1})\cdots R_{{\hat{v}}_{j}}(\phi_{j})$. But $-R_{{\hat{v}}_{1}}(\phi_{1})\cdots R_{{\hat{v}}_{j}}(\phi_{j})=R_{{\hat{v}}_{1}}(\phi_{1}+2\pi )R_{{\hat{v}}_{2}}(\phi_{2})\cdots R_{{\hat{v}}_{j}}(\phi_{j})$. This implies $M_{\hat{m},\hat{n}}^{\mathrm{even}}(F(U))\geq M_{\hat{m},\hat{n}}^{\mathrm{even}}(U)$, and hence, $M_{\hat{m},\hat{n}}^{\mathrm{even}}(F(U))=M_{\hat{m},\hat{n}}^{\mathrm{even}}(U)$. We also have $N_{\hat{m},\hat{n}}(F(U))=N_{\hat{m},\hat{n}}(U)$, etc., similarly.

## Appendix F. Proof of theorem 3.1

Put

$$\delta =\arccos|{\hat{m}}^{T}\hat{n}|\in \left(0,\frac{\pi }{2}\right].$$

Note $N_{-\hat{m},\hat{n}}(U)=N_{\hat{m},\hat{n}}(U)$ by definition. Hence, we shall prove the statement assuming ${\hat{m}}^{T}\hat{n}\geq 0$, which is enough.

First, we give another corollary to lemma 6.2.

Corollary F.1 *For any* α,γ∈R, *and for any* *β*∈[0,*π*],

$$N_{\hat{m},\hat{n}}(U_{\alpha ,\beta ,\gamma }^{\hat{m},\hat{l}})\leq M_{\hat{m},\hat{n}}(U_{\alpha ,\beta ,\gamma }^{\hat{m},\hat{l}})\leq \min\left\{2\left\lceil \frac{\beta }{2\delta }\right\rceil +1,\max_{\alpha^{\prime }\in R}g(\alpha^{\prime },\beta ,\delta )\right\}.$$

Proof The first inequality follows from the definitions of $N_{\hat{m},\hat{n}}$ and $M_{\hat{m},\hat{n}}$. The second inequality immediately follows from corollary 6.3. ▪

It is easy to show, using corollary F.1, that

F 1 $$N_{\hat{m},\hat{n}}(F(U))\leq N_{\hat{m},\hat{n}}(U)\leq \nu +1$$

for any *U*∈SU(2), where *ν*:=⌈*π*/*δ*⌉. But we have $\nu +1\leq N_{\hat{m},\hat{n}}(F(U))$ and, therefore, the equality among all sides of (F 1) for

$$U=\left\{ \begin{array}{ll} R_{\hat{m}}(\pi )R_{\hat{l}}(\pi -\delta ) & \text{if }\nu \text{ is even}\\ R_{\hat{l}}(\pi ) & \text{if }\nu \text{ is odd.} \end{array}\right.$$

Thus, we have proved theorem 3.1 elementarily.

## Appendix G. Procedure for obtaining an optimal decomposition

In proposition 4.4 and remark 4.6, setting *k*=⌈*β*/(2*δ*)⌉, *β*_*j*_=2*δ* for *j*≠*k* and *t*_*j*_=*π*/2 (*j*=1,…,*k*), we have the following special form of (4.6):

G 1 $$R_{\hat{m}}(\alpha )R_{\hat{l}}(\beta )R_{\hat{m}}(\gamma )=R_{\hat{m}}(\alpha ){\left[R_{\hat{n}}(\pi )R_{\hat{m}}(-\pi )\right]}^{k-1}R_{\hat{m}}(-\alpha_{k})R_{\hat{n}}(\theta_{k})R_{\hat{m}}(-\gamma_{k}+\gamma ),$$

where

$$\beta_{k}=\beta -2(k-1)\delta$$

since *β*_*j*_=2*δ* for *j*<*k*.

The analogous special case of (4.12) with *k*′=⌈*β*′/(2*δ*)+1/2⌉, *β*′_*j*_=2*δ* for *j*≠*k*′ and *t*_*j*_=*π*/2 (*j*=1,…,*k*′) is

G 2 $$U=R_{\hat{n}}(\alpha^{\prime }){\left[R_{\hat{n}}(\pi )R_{\hat{m}}(-\pi )\right]}^{k^{\prime }-1}R_{\hat{m}}(-\alpha_{k^{\prime }}^{\prime })R_{\hat{n}}(\theta_{k^{\prime }}^{\prime })R_{\hat{m}}(-\gamma_{k^{\prime }}^{\prime }+\gamma^{\prime }),$$

where

$$\beta_{k}^{\prime }=\beta^{\prime }+\delta -2(k^{\prime }-1)\delta .$$

In particular, if *β*′≤*δ*, this equation becomes

G 3 $$U=R_{\hat{n}}(\alpha^{\prime })R_{\hat{m}}(-\alpha_{k^{\prime }}^{\prime })R_{\hat{n}}(\theta_{k^{\prime }}^{\prime })R_{\hat{m}}(-\gamma_{k^{\prime }}^{\prime }+\gamma^{\prime }).$$

The aim of this appendix is to present a procedure to produce the parameters (angles) of the optimal decomposition having the form of (G 1), (G 2) or (G 3), where interchange of $\hat{m}$ and $\hat{n}$ is allowed.

First, we describe the data format of output decompositions. We shall use a label taking values in {0,1}, where the label 0 indicates that the rightmost factor in the output decomposition is a rotation about $\hat{m}$, and the label 1 indicates the other case. To express sequences of angles efficiently, we introduce the following notation:

G 4 ∧∧jstands for π,−π,…,π,−π,

where the pattern ‘*π*,−*π*’ is repeated *j* times, and

G 5 −∧∧jstands for −π,π,…,−π,π,

where the pattern ‘−*π*,*π*’ is repeated *j* times (j∈Z,j≥0). We put Π={∧∧j∣j∈Z,j≥0}∪{−∧∧j∣j∈Z,j≥0}.

A decomposition is represented as a list of the form

G 6 $$[r_{0},r_{1},\ldots ,r_{N}],$$

where *r*_0_∈{0,1} and $r_{j}\in R\cup \Pi$ for *j*=1,…,*N*. The first entry *r*_0_ denotes the label. The part *r*_1_,…,*r*_*N*_ lists the angles of all factors in a decomposition, where the order is preserved in listing the angles. For example, if the optimal decomposition is $R_{\hat{n}}(\pi /3)R_{\hat{m}}(-\pi /4)$, the output expressing this is [0,*π*/3,−*π*/4]; if the optimal one is $R_{\hat{m}}(\pi /8)R_{\hat{n}}(\pi )R_{\hat{m}}(-\pi )$, the output is [0,*π*/8,*π*,−*π*] (or [0,*π*/8,∧∧1]).

To proceed, we need some definitions. The symbol ⊕ denotes the exclusive or operation (addition in Z/2Z). We define a function reverse as follows: reverse(*s*)=−*s* for s∈R, reverse(*s*)=*s* for *s*∈*Π* and

$$\text{reverse}(r)=[r_{0}⊕1,\text{reverse}(r_{N}),\ldots ,\text{reverse}(r_{1})]$$

for a list *r* of the form (G 6).

We use functions a'(*α*,*β*,*γ*,*δ*) and c'(*α*,*β*,*γ*,*δ*) that return *α*′ and *γ*′, respectively, such that

$$R_{y}(-\delta )R_{z}(\alpha )R_{y}(\beta )R_{z}(\gamma )=R_{z}(\alpha^{\prime })R_{y}(\beta^{\prime })R_{z}(\gamma^{\prime }).$$

We shall not write down algorithms for these functions as it is as trivial as writing down the standard functions a and c in what follows. Below, the functions *b*, *f*, *g* and *σ*_*t*_ defined in §3 will be used.

The core of the procedure consists of the following two functions to represent the above two decompositions, where interchange∈{0,1} is an external variable to be defined outside the functions.

DecompositionOdd (*α*,*β*,*γ*,*δ*,*N*){

 *k*:=(*N*−1)/2; **If** *k*=0, **then return** [interchange, *α*+*γ*]

 *β*_last_:=*β*−2(*k*−1)*δ*;

 (*α*_last_,*γ*_last_,*θ*_last_)^*T*^:=*σ*_*π*/2_(*β*_last_,*δ*); /* *σ*_*t*_ is defined in (4.7) */

 **If** *k*>1, **then**

  **return** [interchange,*α*,∧∧*k*−2,*π*,−*π*−*α*_last_,*θ*_last_,−*γ*_last_+*γ*];

 **else**

  **return** [interchange,*α*−*α*_last_,*θ*_last_,−*γ*_last_+*γ*]; }

DecompositionEven (*α*,*β*,*γ*,*δ*,*N*,*β*′){

 *k*′:=*N*/2;

 *α*′:=a'(*α*,*β*,*γ*,*δ*);

 *γ*′:=c'(*α*,*β*,*γ*,*δ*);

 *β*′_last_:=*β*′+*δ*−2(*k*′−1)*δ*;

 (*α*′_last_,*γ*′_last_,*θ*′_last_)^*T*^:=*σ*_*π*/2_(*β*′_last_,*δ*);

 **If** *β*′>*δ*, **then**

  **return** [interchange,*α*′+*π*, −∧∧*k*′−2, −*π*−*α*′_last_, *θ*′_last_, −*γ*′_last_+*γ*′];

 **else** {

  **If** *β*′=*δ*, **then**

   **return** [interchange,*α*′+*θ*′_last_, −*γ*′_last_+*γ*′];

 **else**

   **return** [interchange, *α*′, −*α*′_last_, *θ*′_last_, −*γ*′_last_+*γ*′]; } }

In what follows, *w*,*x*,*y* and *z* are the parameters to specify

G 7 $$U(w,x,y,z)=\left(\begin{array}{cc} w+iz & y+ix\\ -y+ix & w-iz \end{array}\right)\in \mathrm{SU}(2)$$

as in definition 4.1. Throughout, relations

G 8 $$\hat{m}={\left(m_{x},m_{y},m_{z}\right)}^{T}\;\text{and}\;\hat{n}={\left(n_{x},n_{y},n_{z}\right)}^{T}$$

should be understood.

The following standard functions for converting (*w*,*x*,*y*,*z*) into the Euler angles would not need to be described: a(*w*,*x*,*y*,*z*), b(*w*,*x*,*y*,*z*) and c(*w*,*x*,*y*,*z*), which return α∈R, *β*∈[0,*π*] and γ∈R, respectively, such that

G 9 $$\begin{array}{rl} \sqrt{w^{2}+z^{2}}\cos\frac{\gamma +\alpha }{2} & =w, \end{array}$$

G 10 $$\begin{array}{rl} \sqrt{x^{2}+y^{2}}\sin\frac{\gamma -\alpha }{2} & =-x, \end{array}$$

G 11 $$\begin{array}{rl} \sqrt{x^{2}+y^{2}}\cos\frac{\gamma -\alpha }{2} & =-y \end{array}$$

and

G 12 $$\begin{array}{rl} \sqrt{w^{2}+z^{2}}\sin\frac{\gamma +\alpha }{2} & =-z \end{array}$$

and $\cos(\beta /2)=\sqrt{w^{2}+z^{2}}$, i.e. such that

$$\left(\begin{array}{cc} e^{-i((\gamma +\alpha )/2)}\cos\frac{\beta }{2} & -e^{i((\gamma -\alpha )/2)}\sin\frac{\beta }{2}\\ e^{-i((\gamma -\alpha )/2)}\sin\frac{\beta }{2} & e^{i((\gamma +\alpha )/2)}\cos\frac{\beta }{2} \end{array}\right)=R_{z}(\alpha )R_{y}(\beta )R_{z}(\gamma )=\left(\begin{array}{cc} w+iz & y+ix\\ -y+ix & w-iz \end{array}\right).$$

Similarly, functions $\tilde{a}(m_{x},m_{y},m_{z})$ and $\tilde{b}(m_{x},m_{y},m_{z})$ that return spherical coordinates $\tilde{\alpha }$ and $\tilde{\beta }$, respectively, such that $\left(\cos\tilde{\alpha }\sin\tilde{\beta },\sin\tilde{\alpha }\sin\tilde{\beta },\cos\tilde{\beta })=(m_{x},m_{y},m_{z}\right)$ will be used freely. We also use

$$\mathrm{sign}(x)=\left\{ \begin{array}{ll} 1 & \text{if }x\geq 0\\ -1 & \text{if }x<0\text{,} \end{array}\right.$$

and a function normalised_vprod(*m*_*x*_,*m*_*y*_,*m*_*z*_,*n*_*x*_,*n*_*y*_,*n*_*z*_) that returns $∥\hat{m}\times \hat{n}{∥}^{-1}{\left(\hat{m}\times \hat{n}\right)}^{T}$, recall (G 8).

The following function represents the main step (for obtaining $\tilde{\gamma }$) of the calculation of the SU(2) element associated with $\hat{l}={\left(l_{x},l_{y},l_{z}\right)}^{T}$ and $\hat{m}$ that has been described in appendix A:

G 13 $$\begin{array}{rl} & \tilde{c}(\tilde{\alpha },\tilde{\beta },l_{x},l_{y},l_{z})\\ & \;=\mathrm{sign}(-l_{x}\cos\tilde{\beta }\cos\tilde{\alpha }-l_{y}\cos\tilde{\beta }\sin\tilde{\alpha }+l_{z}\sin\tilde{\beta })\arccos(-l_{x}\sin\tilde{\alpha }+l_{y}\cos\tilde{\alpha }). \end{array}$$

Now we present the procedure, where *w*,*x*,*y* and *z* are the parameters of *U*(*w*,*x*,*y*,*z*) as in (G 7) to be decomposed.

**Procedure for obtaining an optimal decomposition**.

**Inputs**: w,x,y,z∈R with *w*^2^+*x*^2^+*y*^2^+*z*^2^=1; $m_{x},m_{y},m_{z},n_{x},n_{y},n_{z}\in R$ with $m_{x}^{2}+m_{y}^{2}+m_{z}^{2}=1$, $n_{x}^{2}+n_{y}^{2}+n_{z}^{2}=1$ and *m*_*x*_*n*_*x*_+*m*_*y*_*n*_*y*_+*m*_*z*_*n*_*z*_≥0.

**Output**: a list consisting of a label ∈{0,1}, and the angles of all factors in an optimal decomposition.

 interchange:=0;

 $\delta :=\arccos{\hat{m}}^{T}\hat{n}$;

 **If** $b(\hat{m},U(w,x,y,z))<b(\hat{n},U(w,x,y,z))$, **then** {

  (*t*_*x*_,*t*_*y*_,*t*_*z*_):=(*m*_*x*_,*m*_*y*_,*m*_*z*_);

  (*m*_*x*_,*m*_*y*_,*m*_*z*_):=(*n*_*x*_,*n*_*y*_,*n*_*z*_);

  (*n*_*x*_,*n*_*y*_,*n*_*z*_):=(*t*_*x*_,*t*_*y*_,*t*_*z*_);

  interchange:=1; }

 (*l*_*x*_,*l*_*y*_,*l*_*z*_):=normalised_vprod(*m*_*x*_,*m*_*y*_,*m*_*z*_,*n*_*x*_,*n*_*y*_,*n*_*z*_);

 /* Euler angles of SU(2) element associated with $\hat{l}$ and $\hat{m}$ in appendix A */

 $\tilde{\alpha }:=\tilde{a}(m_{x},m_{y},m_{z})$;

 $\tilde{\beta }:=\tilde{b}(m_{x},m_{y},m_{z})$;

 $\tilde{\gamma }:=\tilde{c}(\tilde{\alpha },\tilde{\beta },l_{x},l_{y},l_{z})$;

 /* Main step */

 1. Set $V=R_{z}(\tilde{\alpha })R_{y}(\tilde{\beta })R_{z}(\tilde{\gamma })$ and calculate parameters *w*′,*x*′,*y*′,*z*′ such that

$$U(w^{\prime },x^{\prime },y^{\prime },z^{\prime })=V^{†}U(w,x,y,z)V.$$

 2. Obtain *α*:=a(*w*′,*x*′,*y*′,*z*′), *β*:=b(*w*′,*x*′,*y*′,*z*′), and *γ*:=c(*w*′,*x*′,*y*′,*z*′).

 3. Put *β*′:=*f*(*α*,*β*,*δ*), *β*′′:=*f*(*γ*,−*β*,*δ*), and

$$N:=\min\left\{2\left\lceil \frac{\beta }{2\delta }\right\rceil +1,\;g(\alpha ,\beta ,\delta ),\;g(\gamma ,-\beta ,\delta )\right\}.$$

 4. Do one of the following three processes according to the case:

  Case 1 [ *N*=2⌈*β*/(2*δ*)⌉+1 ]

   **return** DecompositionOdd (*α*,*β*,*γ*,*δ*,*N*);

  Case 2 [ *N*=*g*(*α*,*β*,*δ*) ]

   **return** DecompositionEven (*α*,*β*,*γ*,*δ*,*N*,*β*′);

  Case 3 [ *N*=*g*(*γ*,−*β*,*δ*) ]

   **return** reverse(DecompositionEven (−*γ*−*π*,*β*,−*α*+*π*,*δ*,*N*,*β*′′));

End of the procedure.

## References

[1] Lowenthal F 1971 Uniform finite generation of the rotation group. *Rocky Mt. J. Math.* 1, 575–586. (doi:10.1216/RMJ-1971-1-4-575)

[2] Lowenthal F 1972 Uniform finite generation of the SU(2) and SL(2,R). *Can. J. Math.* 24, 713–727. (doi:10.4153/CJM-1972-067-x)

[3] Wigner EP 1959 *Group theory and its application to the quantum mechanics of atomic spectra.* New York, NY: Academic Press.

[4] Biedenharn LC, Louck JD 1985 *Angular momentum in quantum physics: theory and application.* New York, NY: Cambridge University Press.

[5] D'Alessandro D 2004 Optimal evaluation of generalized Euler angles with applications to control. *Automatica* 40, 1997–2002. (doi:10.1016/j.automatica.2004.06.006)

[6] Koch RM, Lowenthal F 1975 Uniform finite generation of three-dimensional linear Lie groups. *Can. J. Math.* 27, 396–417. (doi:10.4153/CJM-1975-048-0)

[7] Leite FS 1991 Bounds on the order of generation of SO(n,R) by one-parameter subgroups. *Rocky Mt. J. Math.* 21, 879–911. (doi:10.1216/rmjm/1181072975)

[8] Reck M, Zeilinger A, Bernstein HJ, Bertani P 1994 Experimental realization of any discrete unitary operator. *Phys. Rev. Lett.* 73, 58–61. (doi:10.1103/PhysRevLett.73.58) 10056719 10.1103/PhysRevLett.73.58

[9] Boykin PO, Mor T, Pulver M, Roychowdhury V, Vatan F 1999 On universal and fault-tolerant quantum computing: a novel basis and new constructive proof of universality for Shor's basis. In *40th Annu. Symp. on Foundations of Computer Science, 17–19 October 1999*, New York, NY, pp. 486–494. IEEE.

[10] Brezov D, Mladenova C, Mladenov I 2012 Vector decompositions of rotations. *J. Geom. Symmetry Phys.* 28, 67–103.

[11] Brezov D, Mladenova C, Mladenov I 2013 Vector parameters in classical hyperbolic geometry. *J. Geom. Symmetry Phys.* 30, 19–48.

[12] Brezov D, Mladenova C, Mladenov I 2014 A decoupled solution to the generalized Euler decomposition problem in ℝ^3^ and ℝ^2,1^. *J. Geom. Symmetry Phys.* 33, 47–78.

